# Role of restraints on hydrogen atoms in Hirshfeld atom refinement: the case of tri-aspartic acid trihydrate

**DOI:** 10.1107/S2052520625006110

**Published:** 2025-09-05

**Authors:** Ravish Sankolli, Lorraine A. Malaspina, Oleg V. Dolomanov, Peter Luger, Julian J. Holstein, Carsten Paulmann, Wolfgang Morgenroth, Florian Kleemiss, Birger Dittrich, Simon Grabowsky

**Affiliations:** a Departement für Chemie, Biochemie und Pharmazie Universität Bern Freiestrasse 3 3012Bern Switzerland; b OlexSys Ltd, Chemistry Department Durham University Durham DH1 3LE UK; c Fachbereich Biologie, Chemie, Pharmazie, Anorganische Chemie Freie Universität Berlin Fabeckstr. 36a 14195Berlin Germany; d Fakultät für Chemie und Chemische Biologie, Anorganische Chemie, TU Dortmund, Otto-Hahn-Str. 6, 44227Dortmund, Germany; e Fakultät für Mathematik, Informatik und Naturwissenschaften, Erdsystemwissenschaften, Universität Hamburg, Grindelallee 48, 20146Hamburg, Germany; f Institut für Geowissenschaften, Erdsystemwissenschaften, Universität Potsdam, Karl-Liebknecht-Str. 24-25, 14476Potsdam-Golm, Germany; g Institut für Anorganische Chemie, RWTH Aachen, Landoltweg 1a, 52074Aachen, Germany; h Novartis Pharma AG, Novartis Campus, Basel, Switzerland; i Mathematisch-Naturwissen­schaftliche Fakultät Universität Zürich Winterthurerstrasse 190 8057Zürich Switzerland; University of Warsaw, Poland

**Keywords:** hydrogen atoms, restraints, Hirshfeld atom refinement, crystal water

## Abstract

Two polymorphs of l-Asp-l-Asp-l-Asp (DDD) trihydrate as model compounds for biologically important proton-shuttle reactions were investigated with the quantum-crystallographic refinement technique Hirshfeld atom refinement (HAR). With HAR, hydrogen-atom positions are refined freely against the X-ray diffraction data and yield *X*—H bond distances close to those from neutron diffraction. However, the X-ray data of DDD trihydrate do not contain sufficient information to refine anisotropic displacement parameters (ADPs) for the hydrogen atoms, although the data quality is comparable to that of typical oligopeptide or protein datasets, including those with disordered fragments. Therefore, the following restraints were tested for the hydrogen-atom ADPs using *NoSpherA2*/*olex2.refine*: a restraint that approximates isotropic behaviour (ISOR), a restraint that enforces similar movement in any direction (SIMU), a rigid-bond restraint (DELU) and an advanced rigid-bond restraint (RIGU). Although it was found that there is no significant influence of the restraint weights and corresponding ADP values on the *X*—H distances, some recommendations on hydrogen-atom ADP restraint weights to be used in HAR are given. For ISOR, the suggested values are 10 times smaller (stricter) than the default values for non-hydrogen atoms in independent atom model (IAM) refinements, whereas those for RIGU are suggested to be less strict.

## Abstract 2

Restraints for atomic displacement parameters of hydrogen atoms in Hirshfeld atom refinement are tested using two polymorphs of the water-rich l-Asp-l-Asp-l-Asp (DDD) crystal structure.

## 1. Introduction

Water determines the chemistry of all living organisms by its unique properties; it is the essential substance in cells, where water accounts for about 70% of their mass and provides the environment for complex biological processes (Ball, 2008*a* ▸). It can be argued that water is an active biomolecule (Ball, 2008*b* ▸) that plays an essential role in protein structures, functions, and stabilities as well as protein–ligand interactions (Maurer & Oostenbrink, 2019 ▸; Bellissent-Funel *et al.*, 2016 ▸). More specifically, biologically important proton-transfer and proton-shuttle mechanisms very often involve water molecules as mediators (Garczarek & Gerwert, 2006 ▸; Kandori *et al.*, 1995 ▸; Freier *et al.*, 2011 ▸). Despite their undoubted importance in protein/enzyme structures and related biological mechanisms, water molecules are often poorly modelled theoretically (Nittinger *et al.*, 2015 ▸; Ilgü *et al.*, 2021 ▸) and inaccurately determined crystallographically (Carugo & Bordo, 1999 ▸; Schoenborn *et al.*, 1995 ▸) because of their propensity to be dynamic and disordered (Barillari *et al.*, 2007 ▸). Recently, Wlodawer *et al.* (2024 ▸) searched the Protein Data Bank (PDB) and found a surprising lack of water molecules in protein structures even at high resolutions, indicating severe problems with adequate water representations throughout. Nevertheless, X-ray crystallography is still the foremost technique to locate water molecules in protein structures (Nakasako, 2004 ▸; Levitt & Park, 1993 ▸).

Hydrogen-atom positions in intermolecular interactions involving peptide groups and water molecules are decisive for the energetics and mechanisms of proton-transfer reactions; and they are normally not accessible accurately or precisely–or at all–with X-ray crystallography (Woińska *et al.*, 2016 ▸; Dittrich *et al.*, 2005 ▸), especially in soft disordered matter. Therefore, smaller model compounds have been used to experimentally simulate snapshots of proton transfer reactions in crystals (Schiøtt *et al.*, 1998*a* ▸; Schiøtt *et al.*, 1998*b* ▸). For model compounds of proton-transfer reactions involving water molecules, we did not find a similar crystallographic study in the literature. Furthermore, we did not find any water-rich crystal structure of a small peptide with well resolved hydrogen-atom positions searching the Cambridge Structural Database (CSD) and the PDB, considering compounds from dipeptides to oligopeptides with more than two water molecules and refined hydrogen-atom positions anywhere in the structure. There is a theoretical study on ‘proton transfer within short protonated peptides in the presence of water’ (Chen *et al.*, 2011 ▸), but we failed to crystallize such computationally suggested peptides with water molecules for this experimental study. We were then triggered by a study on triaspartic acid (l-Asp-l-Asp-l-Asp, DDD, in the scheme below) in which the authors state that DDD is a suitable model compound to understand ‘the interactions that govern turn formation in the unfolded state of proteins’, but they added that ‘it is still unclear, however, whether short peptides can adopt stable turn structures in aqueous environments in the absence of any nonlocal interactions’ (Duitch *et al.*, 2012 ▸).
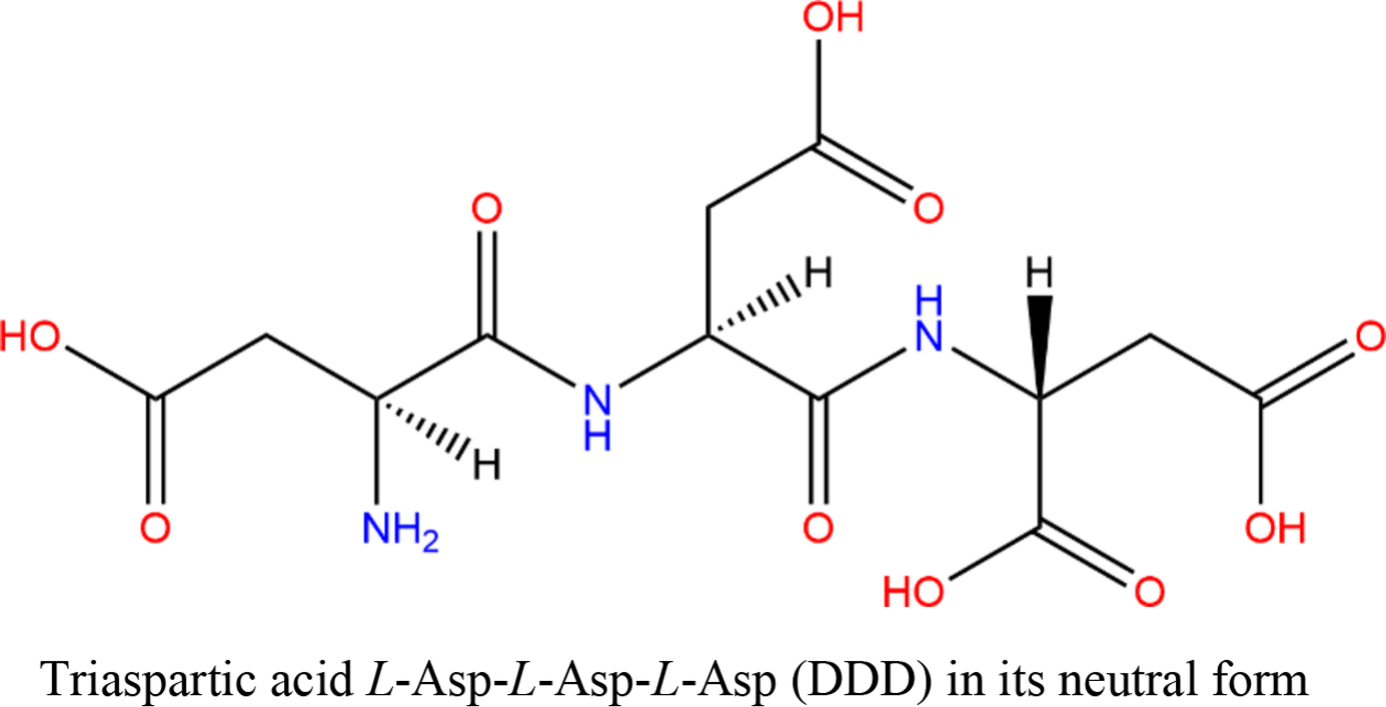
We set out to answer this question experimentally and could crystallize DDD as a trihydrate with 13 mass-% of water with many strong hydrogen bonds between chains of water and with the peptide residues. We subsequently measured two synchrotron X-ray structures at temperatures of 8 K and 100 K, and used modern quantum-crystallographic techniques for the accurate localization of all hydrogen atoms in the crystal structure, especially in the hydrogen bonds.

In quantum crystallography, the non-spherical distribution of the electron density is considered in the calculation of the atomic form factors, *e.g.* by using pseudoatoms in the multipole model (Bentley & Stewart, 1976 ▸; Hansen & Coppens, 1978 ▸) or Hirshfeld atoms (Hirshfeld, 1977 ▸) in Hirshfeld atom refinement (HAR) (Capelli *et al.*, 2014 ▸; Jayatilaka & Dittrich, 2008 ▸). This especially improves hydrogen-atom positions because they have no spherical core but only a covalently bonded valence electron, so the traditionally used independent atom model (IAM) is unsuitable for hydrogen atoms. This means that hydrogen atoms can be localized in organic compounds with X-ray diffraction data as accurately and precisely as with neutron diffraction data when using databases of pseudoatoms (Dittrich *et al.*, 2005 ▸; Dittrich *et al.*, 2017 ▸; Jha *et al.*, 2020 ▸) or HAR (Woińska *et al.*, 2016 ▸; Fugel *et al.*, 2018 ▸; Sanjuan-Szklarz *et al.*, 2020 ▸). For HAR, this is also true if hydrogen atoms are displaced from their donor atoms in strong and short hydrogen bonds (Malaspina *et al.*, 2020 ▸; Malaspina *et al.*, 2021 ▸; Woińska *et al.*, 2014 ▸). Hence, HAR and the related HAR-ELMO or fragHAR approaches could be a solution for protein crystallography whenever hydrogen-atom positions or protonation states play a role in the understanding of the function of the protein/enzyme or the biological mechanism it is involved in (Malaspina *et al.*, 2019 ▸; Bergmann *et al.*, 2020 ▸; Chodkiewicz *et al.*, 2022 ▸). Therefore, HAR is used in this study for DDD trihydrate.

Finding ways of handling problems related to data quality, resolution and disorder is the obstacle to overcome if quantum crystallography is to be used in protein crystallography (Pröpper *et al.*, 2013 ▸). Resolutions need to be ideally better than approximately 1.0 Å (Woińska *et al.*, 2016 ▸). If the data quality is insufficient to locate the hydrogen atoms in the electron-density map, then there is no use in employing quantum-crystallographic techniques (Genoni *et al.*, 2018 ▸) such as HAR to refine hydrogen-atom positions. However, there are many cases where the data quality for polypeptides and smaller proteins is indeed good enough or at least at the borderline to refine hydrogen atoms, but they have nevertheless either been ignored or treated incorrectly in many deposited structures where these techniques have not yet been used (see, for example, Figure 3 in Malaspina *et al.*, 2019 ▸). In cases of borderline data quality, freely HAR-refined *X*—H distances might still be reliable compared to data from neutron diffraction, but freely refined anisotropic displacement parameters (ADPs) can become skewed, oblique, or non-positive definite (NPD). It turns out that the diffraction data of DDD offer such a borderline case, including disorder, and giving rise to skewed and NPD ADPs if refined freely, so our data of DDD serve as a useful example for many polypeptide structures. Such ADPs that look physically unreasonable will in many cases still be identical to those from neutron diffraction within a single combined standard uncertainty and will not deteriorate the accuracy of the refined *X*—H distances (Woińska *et al.*, 2016 ▸; Woińska *et al.*, 2014 ▸). However, NPD ADPs represent a negative volume of probability in real space, which is physically unrealistic and should be avoided (Dittrich *et al.*, 2017 ▸). Such ADPs should be replaced with estimated (Hirshfeld, 1976 ▸; McMullan & Craven, 1989 ▸; Madsen, 2006 ▸) or calculated ones (Flaig *et al.*, 1998 ▸; Roversi & Destro, 2004 ▸; Dittrich *et al.*, 2017 ▸; Lübben *et al.*, 2015 ▸; Madsen *et al.*, 2013 ▸; Malaspina *et al.*, 2020 ▸) or refined only isotropically instead (Malaspina *et al.*, 2020 ▸).

An alternative to the above-discussed options is the use of restraints on ADPs for HAR. However, this option has not existed between the introduction of HAR in 2008 (Jayatilaka & Dittrich, 2008 ▸) and now. We have activated hydrogen-atom ADP restraints in the *olex2.refine* software (Bourhis *et al.*, 2015 ▸) in the framework of the *NoSpherA2* variant of HAR (Kleemiss *et al.*, 2021 ▸), which is part of the *OLEX2* package (Dolomanov *et al.*, 2009 ▸). Restraints introduce additional information into the refinement subject to a probability distribution around a mean value of the restrained parameter defined by its standard uncertainty, also called elasticity. This provides weights or limits within which the parameter can be refined (Waser, 1963 ▸; Müller *et al.*, 2006 ▸); more details in Section 2.2. In this context, we will investigate the following questions.

(i) Are the weights and limits that were originally found for non-hydrogen atoms also applicable for hydrogen atoms or would the default values have to be changed when hydrogen atoms are refined?

(ii) Are the weights and limits that were originally found for IAM also applicable for HAR or would the default values have to be changed when hydrogen atoms are refined in HAR?

(iii) What is the impact of hydrogen-atom ADP restraints on the freely HAR-refined *X*—H bond distances compared to free refinement also of ADPs?

(iv) Can we define recommendations for the routine use of hydrogen-atom restraints in HAR refinements of C—H, O—H and N—H bonds in peptides?

Although none of these questions has been studied systematically before, some of us have used our implementation of the RIGU restraint (see Section 2.2) for hydrogen atoms in HAR previously (Novelli *et al.*, 2021 ▸). After that first study, the option of using restraints also for hydrogen atoms in *olex2.refine* has been left activated and was hence used by quite a few researchers in a short time period, indicating its usefulness. However, it has not been critically assessed, just applied with default weights for non-hydrogen atoms known from IAM (for some examples, see Chocolatl Torres *et al.*, 2021 ▸; Wenger *et al.*, 2021 ▸; Holsten *et al.*, 2021 ▸; Aree *et al.*, 2022 ▸; Koronkiewicz *et al.*, 2022 ▸; Hill *et al.*, 2022 ▸; Sánchez-Sánchez *et al.*, 2024 ▸). We note that by their definition (see Section 2.2), the RIGU and DELU restraints have not been designed for two bonded atoms with significantly different atomic masses, since the light atom, here the hydrogen atom, will vibrate much more in the bond direction than the heavier atom; and the movement of the light atom is much more anharmonic. Hence, a careful consideration of the impact of these restraints on hydrogen atoms is necessary.

Restraints in protein crystallography for non-hydrogen atoms are, of course, a heavily used tool (Steiner & Tucker, 2017 ▸; Bergmann *et al.*, 2022 ▸), but we could not identify any study that uses restraints on hydrogen-atom ADPs although it is in principle possible in the software programs *Phenix* (Headd *et al.*, 2012 ▸), *SHELXL* (although RIGU is deactivated for hydrogen atoms) (Müller *et al.*, 2006 ▸), and *Crystals* (Cooper *et al.*, 2010 ▸). Anyway, it would not make much sense to refine hydrogen-atom ADPs, even if restrained, in these programs because they only support IAM refinements. For other non-spherical atom refinement methods beyond IAM, such as multipole modelling or multipole database applications, only the software *MoPro* allows restraints on hydrogen atoms (Jelsch *et al.*, 2005 ▸). However, the *MoPro* developers and we could not find a study of hydrogen-atom ADP restraints in multipole modelling (Jelsch, 2023 ▸). In general, studies of the impact of restraints on experimental electron-density distributions and accurate geometries in the framework of multipole modelling are scarce (Zarychta *et al.*, 2011 ▸).

## 2. Experimental and theoretical part

### 2.1. Crystallization, synchrotron experiments, and refinement

Tri-aspartic acid was obtained commercially. Crystals were grown by isothermal evaporation from saturated aqueous solutions. Single-crystal X-ray diffraction experiments were carried out at beamlines D3 (DDD trihydrate 8 K) and F1 (DDD trihydrate 100 K) of storage ring DORIS III at HASYLAB/DESY in Hamburg which were equipped with a Huber four-circle diffractometer (D3), a kappa-geometry diffractometer (F1) and marCCD 165 area detectors. Wavelengths of 0.5166 Å (D3) and 0.5600 Å (F1) were chosen. The temperatures were maintained at 8 K/100 K during the measurements by using open helium/nitrogen gas-flow devices. For the 8 K DDD trihydrate dataset, data up to a resolution of *d*_max_ = 0.57 Å were used with a high redundancy of 11.1. However, due to the lowest-symmetry space group *P*1 and geometrical restrictions in the experimental hutch owing to the helijet and the large marCCD detector only a maximum completeness of 93% could be reached. For the 100 K DDD trihydrate dataset, quality indicators (such as *R*_int_, intensity over background) showed that data up to the very high resolution of *d*_max_ = 0.45 Å were useful, although this means low total completeness and low total redundancy of the dataset approaching *d*_max_. Data integration and reduction were carried out with *XDS* (Kabsch, 1988 ▸; Kabsch, 1993 ▸) software. Experimental details are given in Table 1 ▸.

The structures were solved with *SHELXS* (Sheldrick, 2008 ▸), then first refined in the IAM with *SHELXL* (Sheldrick, 2015 ▸) and subsequently with *olex2.refine* (Bourhis *et al.*, 2015 ▸). This last IAM model was used as the starting point for HAR using *NoSpherA2* (Kleemiss *et al.*, 2021 ▸). Within *NoSpherA2*, *Orca* (Neese, 2022 ▸) software was used to calculate the quantum-mechanical electron densities at the B3LYP/def2-TZVPPD level of theory, which were then partitioned into Hirshfeld atoms, Fourier transformed to atomic form factors and stored in .tsc files (Midgley *et al.*, 2021 ▸). These non-spherical atomic form factors were used by *olex2.refine* to perform regular least-squares refinements with restraints, as discussed in the following subsection. The final DDD models were submitted to the CSD and can be obtained free of charge under CCDC deposition numbers 2100425 and 2388831 at https://www.ccdc.cam.ac.uk/structures.

### 2.2. Restraints

Constraints either rigidly relate two or more parameters to each other or assign fixed values to them, so that the number of independent parameters is reduced in refinement (Müller *et al.*, 2006 ▸). In contrast to constraints, restraints do not reduce the number of refinable parameters but include additional observations into the refinement from independent experiments or calculations, so that the equation Δ to be minimized during the least-squares procedure obtains an additional term (Müller *et al.*, 2006 ▸; Bourhis *et al.*, 2015 ▸):
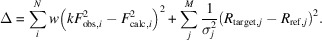
The first sum is the usual minimization term for the weighted observed and calculated difference of structure factor magnitudes, where *k* is the scale factor. The second sum contains the standard uncertainty *σ_j_* for each restrained parameter *R_j_*. Specifically, 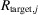 is the target value of the restrained parameter obtained separately and 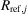 is its refined value, whereby there can be more than one value *R_j_* with its associated *σ_j_* per restraint. *σ_j_* defines the limits within which the parameter is allowed to vary during the refinement and consequently 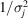 is the weight of the restraint *R_j_*.

All restraints used here refer to their implementation in *olex2.refine* (Bourhis *et al.*, 2015 ▸). A normalization factor is determined to place the restraints on the same scale as the average residual [for details, see Section 4, especially equation 25, in Bourhis *et al.* (2015 ▸)]. The four-letter abbreviations used conform to the corresponding *SHELXL* keywords. In the final DDD models that are discussed in Section 3.1, some *X*—H distance restraints were used in addition to ADP restraints. Such distance restraints (SADI for NH_3_ and CH_2_ groups as well as DFIX and DANG for the disordered water molecules) are not further discussed in this paper. Here, we concentrate on hydrogen-atom ADP restraints and how they impact on freely HAR-refined *X*—H distances. There are four types of ADP restraints that we discuss and review briefly below (Müller *et al.*, 2006 ▸; Bourhis *et al.*, 2015 ▸; SHELXL, 2023 ▸).

#### 2.2.1. ISOR

**Figure 1:**
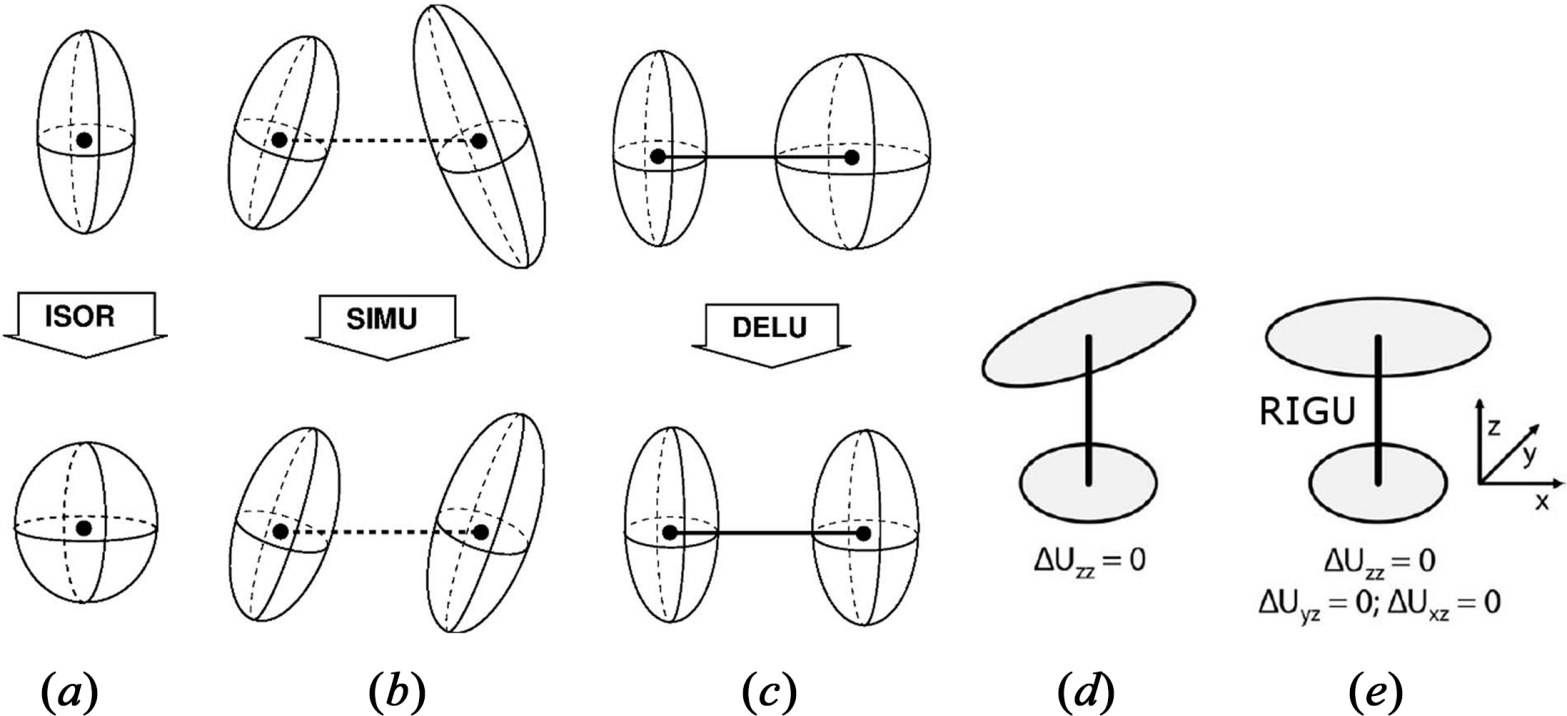
Effect of ADP restraints. (*a*) ISOR: restraint to induce approximately isotropic behaviour; (*b*) SIMU: restraint to align the direction of movement of two bonded atoms; (*c*) DELU: restraint to enforce Hirshfeld’s rigid bond condition; (*d*) illustration of a situation where the rigid bond test is fulfilled (adhering to DELU), but the directions of movements are misaligned in a perpendicular plane; (*e*) RIGU: enhanced rigid bond restraint in which the movements in the two perpendicular planes are also aligned. Adapted from Schneider (1996 ▸), with permission from T. R. Schneider, and from Thorn *et al.*, (2012 ▸), under the general licence agreement of IUCr journals.

This restraint changes the shape of the ADP to be closer to isotropic [Fig. 1 ▸(*a*)]. There are two different default values for the related σ, derived for non-hydrogen atoms in IAM refinements: 0.2 Å^2^ for terminal atoms and 0.1 Å^2^ for all other atoms. For our HAR application, we have varied σ from a value of 0.00001 Å^2^, strictly enforcing a spherical shape, to a maximum value of 1.0 Å^2^, which has shown to be loose enough so that those hydrogen-atom ADPs that become NPD when freely refined become also NPD under the restraint. We have observed the variation of the *X*—H bond lengths upon changing the shapes of the hydrogen ADPs within this range of σs.

#### 2.2.2. SIMU

The assumption behind this restraint is that two atoms bonded to each other move in similar directions with a similar amplitude [Fig. 1 ▸(*b*)]. For non-hydrogen atoms, a distance threshold below 1.7 Å is used to define if two atoms are bonded to each other. The corresponding σ for the non-hydrogen ADP components *U*_*ij*_ of the two atoms to be harmonized is 0.08 Å^2^ for terminal atoms and 0.04 Å^2^ for all other atoms. SIMU includes three different modifications or limits (distance, direction, and amplitude) that are rather strong, so it is difficult to include it in a systematic analysis and we have, hence, not studied it in Section 3. However, we show in which case it must be used with a weak weight for hydrogen atoms in Section 3.2.4.

#### 2.2.3. DELU

This ‘rigid-bond restraint’ is based on the Hirshfeld rigid-bond condition (Hirshfeld, 1976 ▸) that two atoms bonded to each other move in a similar manner along the bond direction [Fig. 1 ▸(*c*)]. There are two σ values for DELU, derived for non-hydrogen atoms in IAM refinements: for directly bonded atoms (1,2-distance) the default value is σ = 0.01 Å^2^, and for two atoms that are bonded via a third common atom (1,3-distance) the value is also 0.01 Å^2^. Here, we have studied both 1,2- and 1,3-scenarios for hydrogen atom ADPs in HAR, varying σ in the range from 0.0001 Å^2^ (strict restraint) to around 0.05 Å^2^ (weak restraint), observing changes in the freely HAR-refined *X*—H bond lengths. Importantly, the application of DELU alone on some of the NPD hydrogen atom ADPs in DDD did not lead to them becoming positive definite. Therefore, we combined DELU always with a weak ISOR restraint. The exact values of these underlying restraints are given in the supporting information (Table S1) as discussed in the last paragraph of this section.

#### 2.2.4. RIGU

Fig. 1 ▸(*d*) visualizes how the rigid-bond condition can be fulfilled between two atoms, but the alignment of the movements in a plane perpendicular to the bond are still unphysical. To restrain such refinement outcomes further, an enhanced rigid-body restraint was developed (Thorn *et al.*, 2012 ▸) that does additionally enforce similar movements in the two other directions [Fig. 1 ▸(*e*)]. This means that each application of RIGU in fact produces three restraints at once, so per definition RIGU is harder than DELU and often supersedes it. As for DELU, there are two σ values, derived for non-hydrogen atoms in IAM refinements: for 1,2-distances, the default value is σ = 0.004 Å^2^, and for 1,3-distances the value is also 0.004 Å^2^, so the default use of RIGU leads to a much harder restraint than the use of DELU with its default σ values. Here, we have studied both 1,2- and 1,3-scenarios for hydrogen atom ADPs in HAR, varying σ in the range from 0.0001 Å^2^ (strict restraint) to around 0.05 Å^2^ (weak restraint), observing changes in the freely HAR-refined *X*—H bond lengths. And, as for DELU, we combined it with a weak ISOR restraint.

#### 2.2.5. Testing the restraints on hydrogen atoms

For testing the hydrogen-atom ADP restraints discussed above, we first refined the 8 K crystal structure of DDD in HAR with mild ISOR restraints on hydrogen atoms where necessary to avoid NPD ADPs, and with isotropic hydrogen atoms at the water molecules including DFIX and DANG distance restraints. We consider this the standard structure that was saved as a starting point for testing. Subsequently, only the hydrogen-atom restraints in one of the different functional groups of DDD (–COOH, –CH, –CH_2_ –NH and –NH_3_^+^) were modified, while all other parameters were kept unmodified, before re-refining the structure. For subsequent refinements with the next higher σ-value of a particular restraint, the starting point was always the standard structure, not the one from the previous refinement. This resulted in hundreds of individual refinements. We modified the *olex2.refine* output so that the resulting *X*—H bond distances were always given with five decimal places to allow better statistical evaluation. The results are discussed in sections 3.2.1 to 3.2.3. From these results, we extracted the optimum σ’s of all hydrogen atom ADP restraints for DDD and derived recommendations for new default values (Section 3.2.4). Finally, we applied these values to both the 8 K and 100 K DDD crystal structures for a final optimal refinement to analyse the water-rich hydrogen-bonding network of DDD (Section 3.1). Importantly, application of the weights derived from the 8 K structure to the 100 K structure is an independent test of the usefulness and generalizability of the recommended values.

## 3. Results and discussion

### 3.1. The crystal structures of triaspartic acid trihydrate

**Figure 2:**
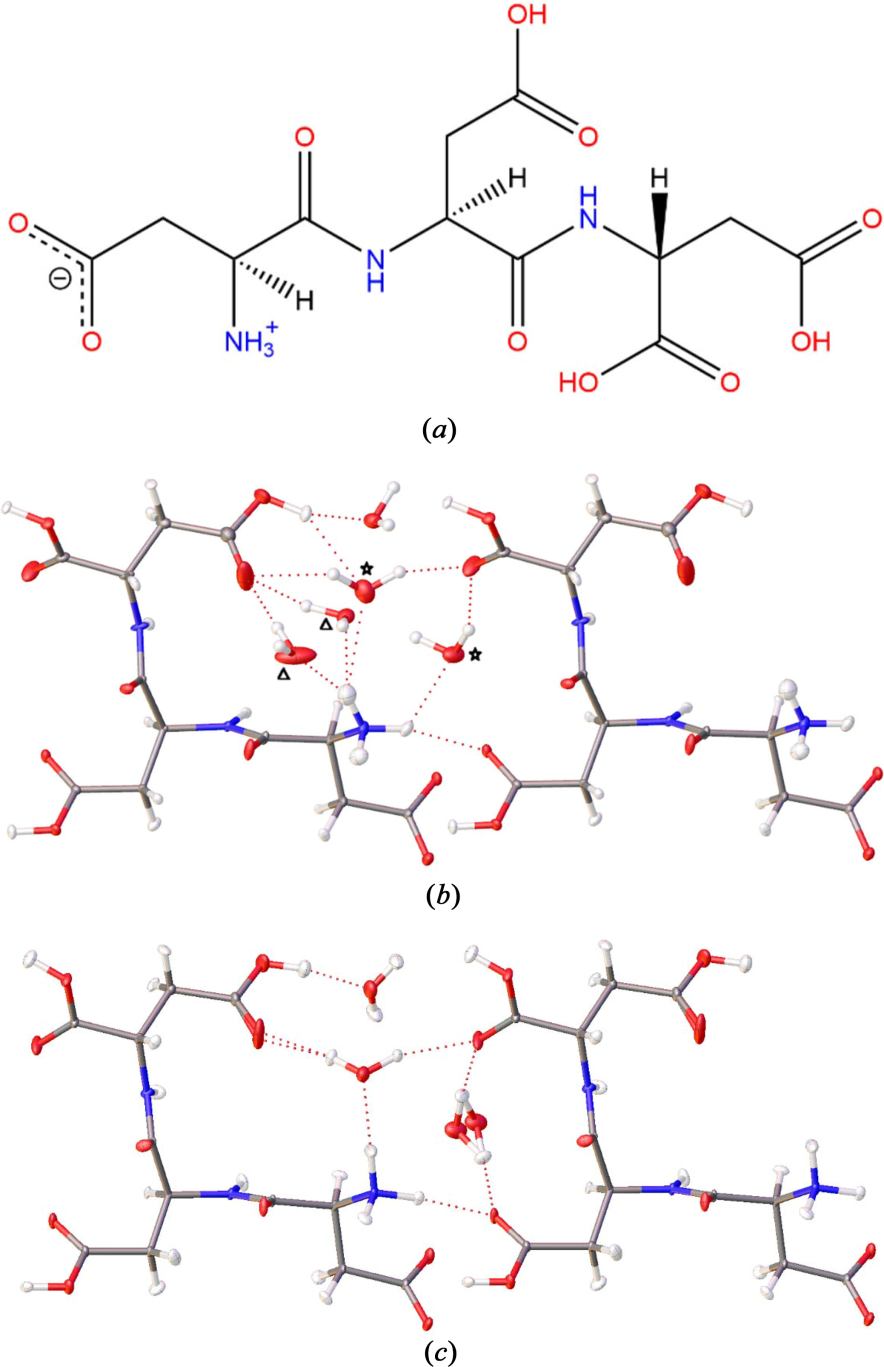
(*a*) Molecular structure of DDD showing the zwitterion configuration found in the crystal structure. (*b*) HAR-refined structure of tri-aspartic acid trihydrate at 8 K; one asymmetric unit plus an additional DDD tripeptide to illustrate the hydrogen bonding network. There are two pairs of mutually exclusive disordered water molecules [pair * with refined occupancies 0.13/0.87 (O41/O51), and pair Δ with 0.25/0.75 (O21/O31)]. Hydrogen atoms in the disordered water were refined isotropically; all others anisotropically with restraints where necessary (Section 3.2.4). (*c*) HAR-refined structure of tri-aspartic acid trihydrate at 100 K; one asymmetric unit plus an additional DDD tripeptide to illustrate the hydrogen bonding network. Disorder is shown. All hydrogen atoms were refined anisotropically with restraints, where necessary. All ADPs at a 50% probability level, isotropic ones at arbitrary scale. All atom labels are given in Fig. S1. Pictures generated with *OLEX2*.

We will first discuss the crystal structures of DDD trihydrate in the final HAR models. These final HAR models make use of a variety of hydrogen-atom restraints with their optimal weights, as we derive and discuss in detail in Section 3.2. Here, we first focus on the structural and chemical aspects. Fig. 2 ▸ shows that DDD crystallizes as a zwitterion with the charged functional groups being located in direct vicinity of each other at the N-terminus of the molecule. It is unusual for tripeptides that the C-terminus is still protonated, which is here a consequence of the availability of three additional carboxyl groups provided by the aspartic acid building blocks. As this would allow a great variety of possible structural motifs and conformations in different deprotonation modes, it is understandable that DDD was used as the most suited model compound to investigate small peptide turn and fold behaviour theoretically (Duitch *et al.*, 2012 ▸). In the experimental DDD structure elucidated in this study, the energetically most favourable conformation is indeed a turn structure leading to a horseshoe shape of the peptide main framework (Fig. 2 ▸). The folded conformation of DDD is enabled by water molecules bridging the ammonium group at the N-terminus with the carboxyl group at the C-terminus with strong hydrogen bonds (Fig. 2 ▸). At the same time, the conformation is stabilized by a water molecule bridging two of the protonated carb­oxy­lic acid groups at the backend of the horseshoe shape of the peptide and connecting them to the ammonium group as well. This finding underlines the importance of water molecules and hydrogen atoms for an understanding of peptide conformations; and consequently, the importance of describing them accurately in X-ray crystallographic studies.

Both the 8 K and 100 K DDD structures constitute trihydrates, because the water disorder pattern in the 8 K crystal structure includes two pairs of mutually exclusive water molecules. Either water molecule O41 or O51 in pair * can be present in the same unit cell; similarly either O21 or O31 in pair Δ [Fig. 2 ▸(*b*)]. Interestingly, the difference between the 8 K and 100 K structures is not only an order–disorder transition, nor can it be attributed entirely to a cooling effect, although the unit-cell volume shrinks in the expected order of magnitude from 456.37 (10) Å^3^ at 100 K to 443.3 (4) Å^3^ at 8 K. The two different structures are thus considered as polymorphs of each other because of the existence of different water molecules in clearly different sites: the water molecule pair depicted with Δ in Fig. 2 ▸(*b*) is absent in the 100 K structure [Fig. 2 ▸(*c*)] altogether. Instead, both water molecules that form the disordered pair * in the 8 K structure are each fully occupied in the 100 K structure. Consequently, some of the main hydrogen bonds that stabilize the peptide conformation are different between the two polymorphs (Table S2; atom labelling Fig. S1). Whereas the few shortest hydrogen bonds involving peptide N—H or O—H donor groups are the same between both polymorphs [O4—H4⋯O6 is the shortest in both, connecting one of the protonated with the deprotonated carboxyl­ate group intermolecularly; 8 K: H4⋯O6 = 1.53 (2) Å, O4⋯O6 = 2.5284 (19) Å, O4—H4⋯O6 = 173 (3)°, 100 K: H4⋯O6 = 1.575 (12) Å, O4⋯O6 = 2.5310 (6) Å, O4—H4⋯O6 = 164.5 (17)°], some of the strongest hydrogen bonds involving water molecules are different. Water O31, the major component of disorder pair Δ, dominates in the 8 K structure [H1*C*⋯O31 = 1.84 (3) Å, N1⋯O31 = 2.703 (2) Å, N1—H1*C*⋯O31 = 151 (3)°, and H31*A*⋯O9 = 1.701 (17) Å, O31⋯O9 = 2.645 (2) Å, O31—H31*A*⋯O9 = 167 (2)°], whereas in the 100 K structure the dominating water molecule is the central non-disordered one that connects two carb­oxy­lic acid groups with the ammonium group [H1*C*⋯O21 = 1.798 (17) Å, N1⋯O21 = 2.8033 (6) Å, N1—H1*C*⋯O21 = 158.0 (2)° and H21*A*⋯O9 = 1.815 (10) Å, O21⋯O9 = 2.7398 (17) Å, O21—H21*A*⋯O9 = 160.5 (13)°]. This water molecule would correspond to O41 in the 8 K structure, which is the minor disorder component of pair *, and its interactions are not even listed among the strongest 12 hydrogen bonds in this structure. This describes the major difference between the two polymorphs.

**Figure 3:**
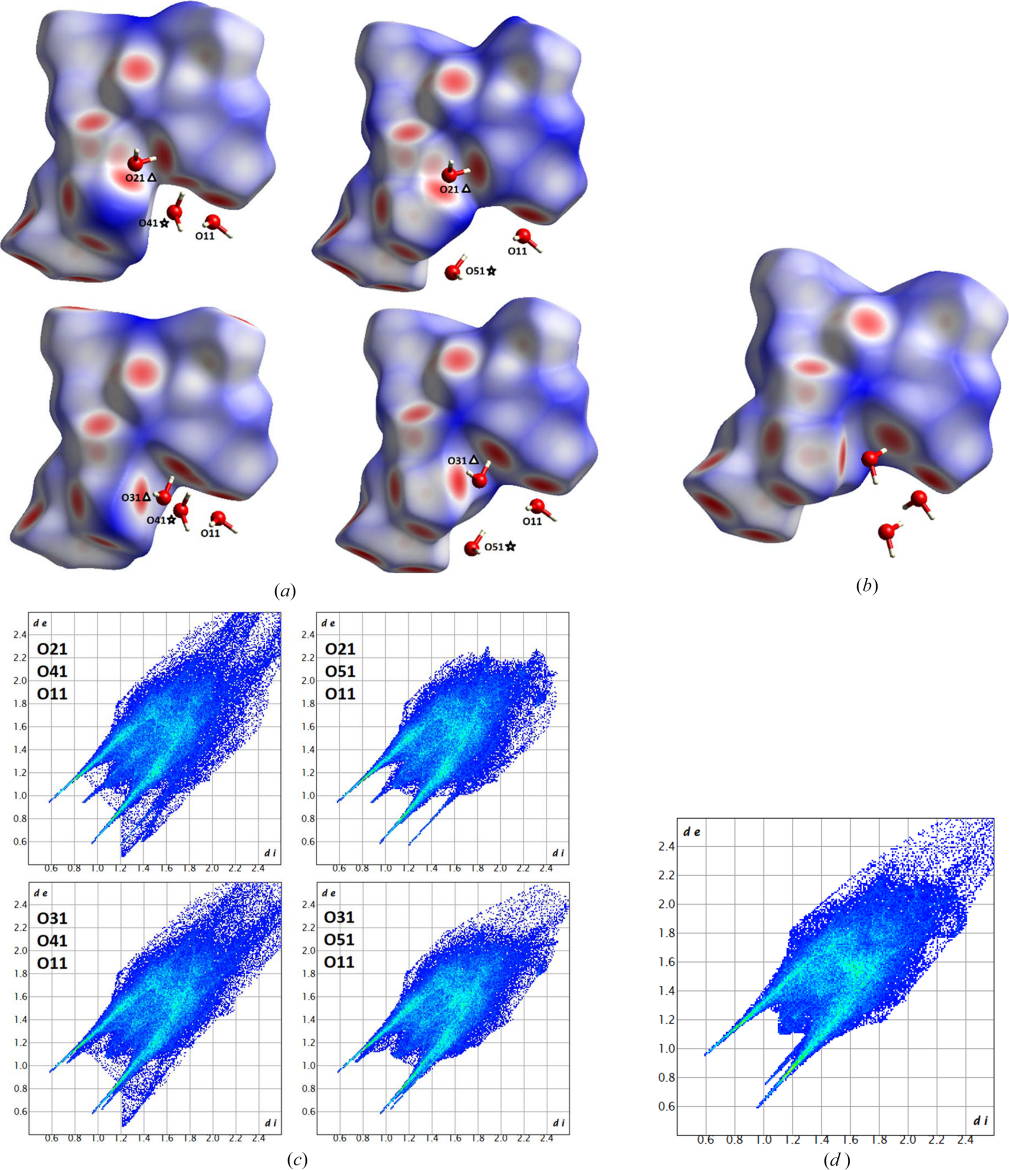
(*a*) and (*b*) Hirshfeld surfaces colour coded with the property *d*_norm_. Red areas depict close contacts, here identical with hydrogen bonding, blue areas distant contacts. Labelled from *d*_norm_ = −0.83 (red) to 0 (white) to 1.54 (blue). All four different water environments of the DDD trihydrate at 8 K are shown, resulting from the water disorder (*a*). The water environment using the main disorder components of the 100 K structure is shown in (*b*). The corresponding fingerprint plots are given in (*c*) and (*d*). Generated with the software *CrystalExplorer* (Spackman *et al.*, 2021 ▸).

In total, there are four different possible water environments that statistically occur in the unit cells of the 8 K structure, and one in the 100 K structure (here, a minor, possibly dynamic, disorder is neglected). They are all plotted in Figs. 3 ▸(*a*) and 3 ▸(*b*) with a Hirshfeld surface (Spackman & Jayatilaka, 2009 ▸) around the DDD molecule to highlight the interactions caused by the water molecules. At first glance, the Hirshfeld surfaces and the distribution of red spots that represent close contacts (here all hydrogen bonds) look very similar. However, note for the 8 K structure how the red colour and shape distribution is different when O21 bridges C- and N-terminus (upper row) compared to O31 (lower row). O21 is bonded to the ammonium group with two contacts of less intense colour than O31 where it is only one contact. In addition, the role of O41 bonding to the same carboxyl oxygen atom as O21/O31 is different to the role of O51 which is not in contact with the same C-terminus carboxyl group. The Hirshfeld surface with contact spots for the 100 K structure [Fig. 3 ▸(*b*)] rather resembles those with O31 present.

The fingerprint plots (Spackman & McKinnon, 2002 ▸) in Figs. 3 ▸(*c*) and 3 ▸(*d*) confirm that all five networks are similar yet different. The occurrence and distribution of spikes (shortest contact, here hydrogen bonds) is not identical among any of the five. Another interesting finding is that the packing realized with water molecules O11, O21, and O51 in the 8 K crystal structure is the only one that avoids void areas, which are depicted by a diffuse region of contact points at the upper right (long distances) of the plot. When comparing torsion angles of the main peptide chain that forms the horseshoe-like fold motif, it turns out that they are the same within a few standard uncertainties (s.u.s) (values present in the CIFs). This means that all water environments stabilize the same peptide folding. However, there are significant conformational differences in the two polymorphs with respect to the rotation of the three carboxyl­ate/ carb­oxy­lic acid groups that are sticking out towards the backend of the main folded chain. The rotation of the deprotonated carboxyl­ate group at the N-terminus relative to the main chain is described by a torsion angle difference of 7.1° in O6—C8—C7—C1 [8 K: 125.74 (7)°, 100 K: 118.66 (4)°], and the rotation of the C-terminal protonated carb­oxy­lic acid group relative to the main chain is described by a torsion angle difference of 7.6° in O3—C6—C5—N3 [8 K: −22.75 (11)°, 100 K: −15.19 (5)°].

During the refinement of the 8 K structure, it was found that different converged structure models with slightly better *R* values can be obtained by freely refining the occupancies of the water molecules. They would add up to only 90–95% of water occupation in the unit cell. In this case, the 8 K and 100 K structures are not strictly polymorphs of each other, but non-stoichiometric hydrates (Authelin, 2005 ▸). Another aspect is that the average structure and the resulting coordinates refined by X-ray diffraction do not necessarily correspond to local or global energy minima. We therefore molecule-in-cluster optimized (Dittrich *et al.*, 2020 ▸) three DDD archetype structures (Dittrich *et al.*, 2024 ▸) from two sets of starting coordinates taken from the 8 K structure with differing carboxyl­ate and water positions, and a third set corresponding to the major occupancy atoms of the 100 K structure. The GFN2-xTB method was used in this context (Bannwarth *et al.*, 2019 ▸). This procedure enabled identification which of the four possible hydrogen-bond patterns in the 8 K structure are corresponding to energy-minimized states. Fig. S2 shows an overlay of the two archetype structures that contribute to the 8 K structure, thereby reproducing its average fully occupied trihydrate structure found experimentally. This, in turn, supports the interpretation of the 8 K and 100 K structures to be polymorphs.

### 3.2. Evaluation of hydrogen-atom restraints in HAR

When the DDD trihydrate crystal structures are refined in HAR without any restraints or constraints on the hydrogen atoms, several of the hydrogen-atom ADPs become skewed and oblique or turn NPD. For the main DDD peptide molecule, this is especially problematic for the N—H and O—H bonds. Hence, these are suitable structures for testing the impact of a variation of the restraint σs on the *X*—H bond distances. In the next three subsections, ISOR, DELU and RIGU are scrutinized based on the 8 K structure, and, subsequently, the values that were found to be optimal are tested on the 100 K structure independently. For details on the large number of underlying individual refinements, see Section 2.2.

#### 3.2.1. ISOR

**Figure 4:**
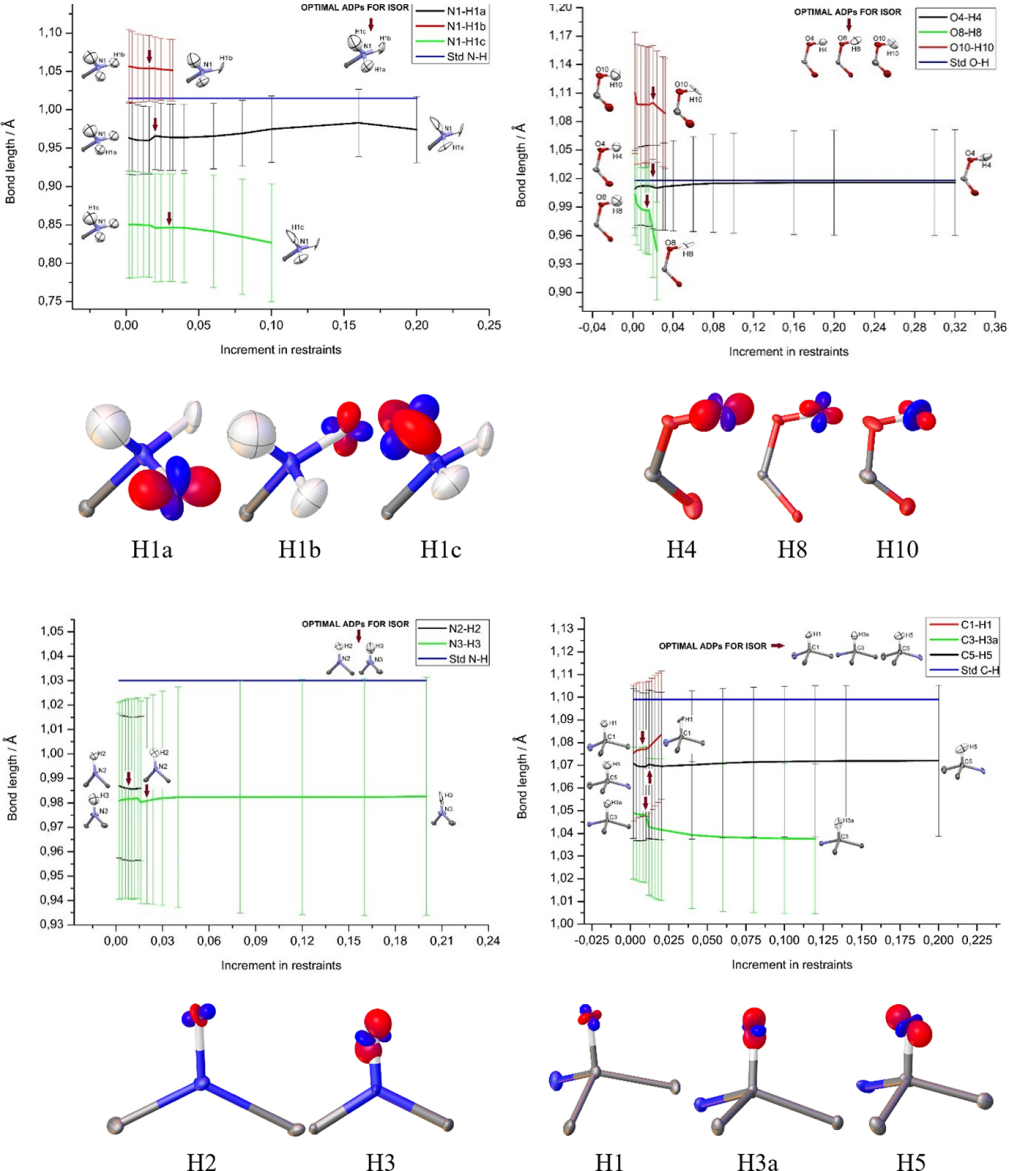
Refined *X*—H bond lengths (Å) versus ISOR restraint σs (Å^2^) for –NH_3_^+^, –COOH, –NH, and –CH functional groups. Vertical bars are the s.u.s upon least-squares refinement. The respective reference *X*—H bond distances from neutron diffraction (Allen & Bruno, 2010 ▸) are given as blue lines. The visual appearance of the ADPs for the two extreme choices of ISOR σs are given as inserts in the plots. Red arrows show the value chosen as optimum compromise between flexibility and acceptable shape of the ADPs, given above the plots. These values are 0.02/0.016/0.03 Å^2^ for H1*A*/H1*B*/H1*C*, 0.02/0.03/0.02 Å^2^ for H4/H8/H10, 0.008/0.02 Å^2^ for H2/H3, and 0.008/0.01/0.012 Å^2^ for H1/H3*A*/H5. Below each plot, corresponding differences between two RMSD surfaces are visualized in the PEANUT style (Hummel *et al.*, 1990 ▸), plotted in *OLEX2*. The difference is always between the optimal value and the biggest value of the restraint (restrained minus unrestrained), blue = positive, red = negative, same scale.

Fig. 4 ▸ shows the N—H, C—H and O—H bond-distance dependence on the ISOR restraint. The loosest ISOR value on the right-hand sides of the plots represents the empirically found situation when the hydrogen atom ADP is just not NPD anymore. Towards the left-hand sides, the restraint becomes more and more strict, until the ADPs are forced to be spherical. The result is that, for all bonds, the change in bond distance is within the s.u. of this bond from the variance-covariance matrix of the refinement; and for most of the bonds the impact is so small that the line is horizontal. Only for O8—H8 and O10—H10, the difference in bond distance between the two extreme choices of the ISOR restraint is more than 0.02 Å. This means that the introduction of the restraint does not improve the bond distances towards the reference values from neutron diffraction (blue lines). And unlike in other studies with higher-quality data (Woińska *et al.*, 2014 ▸; Woińska *et al.*, 2016 ▸; Fugel *et al.*, 2018 ▸; Sanjuan-Szklarz *et al.*, 2020 ▸; Malaspina *et al.*, 2020 ▸; Malaspina *et al.*, 2021 ▸), only some of the *X*—H bonds in the HAR model of DDD match the neutron references within a single s.u. value.

Starting from the two extreme (NPD or isotropic) hydrogen-atom ADPs, their variation has no significant effect on the *X*—H bond distances for DDD: within all s.u.s of the distances and the ADP values, an NPD ADP is statistically as meaningful as a spherical one or as a restrained/constrained ellipsoidal one that is physically correct. This in turn means that if a physically meaningful ADP is preferred, there is no indication that the default values for non-hydrogen atoms found for IAM (0.1 and 0.2 Å^2^, see Section 2.2) are detrimental or at all impactful for the HAR refinement procedure if they fulfil their purpose (making the shape physically meaningful). The values we have chosen here to be optimal (red arrows, see caption of Fig. 4 ▸) are in that sense arbitrary. The criterion used for their choice was that the related *X*—H bond distance should be closest to the reference neutron value whereby the ADPs should look visually acceptable, and the *R* value should be lowest. It turns out that all the chosen values are smaller (stricter) by at least a factor of 10 than the default ones for non-hydrogen atoms. With the default values, the skewness and obliqueness in the ADP shapes cannot be cured.

Below each graph, differences between two root-mean-square displacement (RMSD) surfaces are visualized according to the PEANUT philosophy and style (Hummel *et al.*, 1990 ▸), here implemented in *OLEX2*. It shows the difference between the displacement modes of the skewed/oblique ADPs (right hand side of the graphs) and the optimal restrained ADPs. Since the restrained ones are made to be more spherical, quadrupolar features are expected for each difference RMSD surface. Otherwise, there is no correlation between directions or magnitudes of the different hydrogen displacements that would allow conclusions about systematic information that is present in the unrestrained hydrogen ADPs. Simply, the data quality is not high enough to allow for physically meaningful freely refined hydrogen ADPs, so that restraining them is justified. Alternative ways to obtain physically meaningful ADPs are discussed as outlook in the conclusions section.

#### 3.2.2. DELU

**Figure 5:**
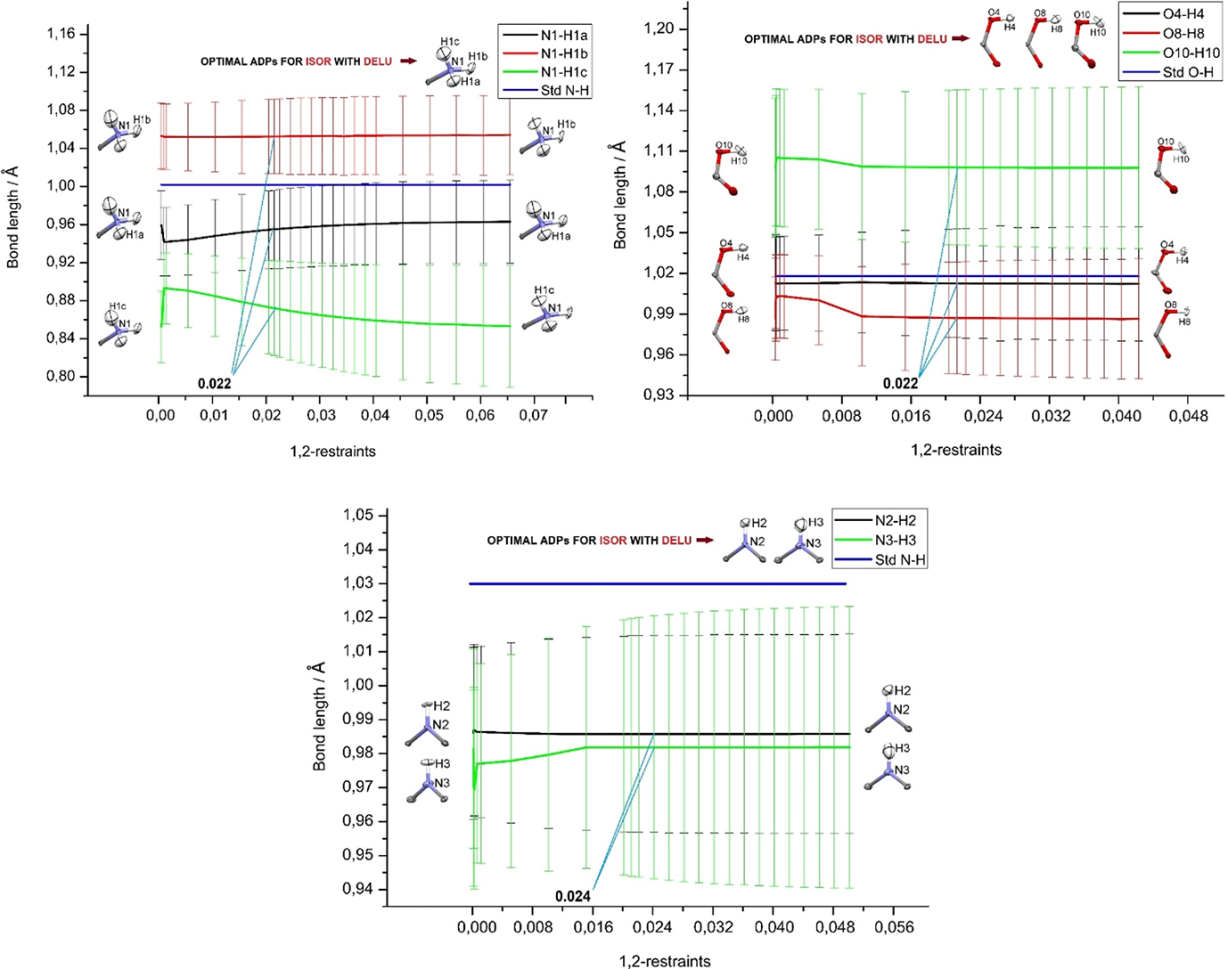
Refined *X*—H bond lengths (Å) versus DELU 1,2-restraint sigmas (Å^2^) for –NH_3_^+^, –COOH, and –NH functional groups, under a mild ISOR restraint. The respective reference *X*—H bond distances from neutron diffraction (Allen & Bruno, 2010 ▸) are given as blue lines. The visual appearance of the ADPs for the two extreme choices of DELU sigmas are given as inserts in the plots. The values chosen as optimum compromise between flexibility and acceptable shape of the ADPs are given in the plots (in Å^2^), and the corresponding ADP shapes above the plots. Similar plots for the DELU 1,3-restraint sigmas are given in Fig. S8.

Fig. 5 ▸ shows the findings for the DELU restraint plotted against the *X*—H bond distances. Towards the left-hand sides, the restraint becomes more and more dominant. Similar findings hold as for the ISOR restraint in the previous subsection: there is no significant impact of the weight of the restraint on the refined *X*—H bond distances within a single s.u., with only a few variations above 0.02 Å when very small (strict) values of the restraint are chosen. No systematic improvements towards the neutron reference values are observed. The optimal values (0.022, 0.022, 0.024 Å^2^) were chosen to be the smallest (strictest) values when the graphs are becoming virtually horizontal and the *R* value lowest, which is, however, a tiny effect. For the 1,3-restraint values, there is no impact on the *X*—H distances at all (see Fig. S8). This means that there is no indication for DELU either that the default values (0.01 Å^2^ for both 1,2- and 1,3-distances) would have any (negative) impact on the HAR refinement.

#### 3.2.3. RIGU

**Figure 6:**
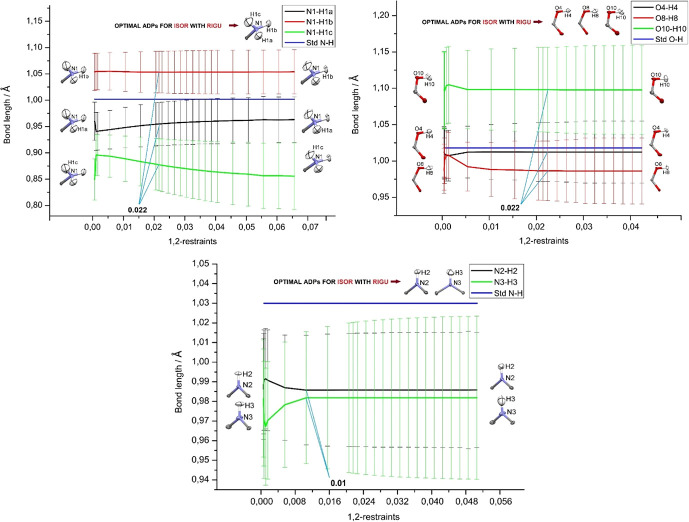
Refined *X*—H bond lengths (Å) versus RIGU 1,2-restraint sigmas (Å^2^) for –NH_3_^+^, –COOH, and –NH functional groups, under a mild ISOR restraint. The respective reference *X*—H bond distances from neutron diffraction (Allen & Bruno, 2010 ▸) are given as blue lines. The visual appearance of the ADPs for the two extreme choices of RIGU sigmas are given as inserts in the plots. The values chosen as optimum compromise between flexibility and acceptable shape of the ADPs are given in the plots (in Å^2^), and the corresponding ADP shapes above the plots. Similar plots for the RIGU 1,3-restraint sigmas are given in Fig. S9.

For the improved rigid-bond restraint RIGU, summarized in Fig. 6 ▸, there are very few differences to the DELU restraint discussed in the previous subsection. The only notable difference is that the impact of the restraint on the refined *X*—H distances is larger at strict restraint values (left hand-side) than for DELU, however, always still within a single standard deviation for the refined *X*—H bond. As for DELU, the differences between the distances of the three individual N—H bonds in the ammonium group are much bigger than the differences of the distances at the two extreme ends of the restraint values in the graphs. Again, the optimal values (0.022, 0.022, 0.010 Å^2^) were chosen to be the strictest values when the graphs are becoming virtually horizontal with the lowest *R* values of the model. For the 1,3-restraint values, there is no impact on the *X*—H distances at all (Fig. S9). This means that there is no indication for RIGU either that the default values would have any (negative) impact on the HAR refinement, but the values we have chosen are always bigger than the default values (see discussion in next subsection).

#### 3.2.4. Discussion and application of hydrogen ADP restraints

The findings of the previous subsections were used to design the final HAR models of DDD trihydrate for both the 8 K and 100 K datasets, whose structural results were discussed in Section 3.1. For ISOR, these values are 0.02/0.016/0.03 Å^2^ for H1a/H1b/H1c, 0.02/0.03/0.02 Å^2^ for H4/H8/H10, 0.008/0.02 Å^2^ for H2/H3, and 0.008/0.01/0.012 Å^2^ for H1/H3a/H5. However, as it was shown in Section 3.2.1 that these numbers are not having a strong effect for X-ray diffraction data, we can average them to get a recommended value for ISOR for hydrogen atoms in organic compounds: 0.02 Å^2^. This value is smaller by a factor of 10 than the default value for terminal non-hydrogen atoms (0.2 Å^2^), which means that hydrogen atoms need a stricter restraint for their ADPs to become reasonably shaped when they started off as NPD ADPs. However, the default of 0.2 Å^2^ will have no negative impact on the *X*—H bond distances, so it can be used, but may not be as effective on the ADP shapes as for non-hydrogen atoms.

For DELU and RIGU, the chosen values are 0.010, 0.022 and 0.024 Å^2^ across all bond types. The default values are 0.01 Å^2^ for DELU, but 0.004 Å^2^ for RIGU. The former approximately agrees with the values we have chosen as optimal, so for DELU we recommend to use the non-hydrogen-atom IAM defaults of 0.01 Å^2^ (1,2- and 1,3-restraints) also for hydrogen atoms in HAR. However, for RIGU the default value is stricter than we deem necessary in most cases to have a significant effect on the shape of the hydrogen-atom ADPs. We therefore rather recommend the same value as for DELU (0.01 Å^2^ for 1,2- and 1,3-restraints), but since restraint values and bond distances do not correlate for DDD and the s.u.s from the refinement are large, there is no statistically more or less reasonable value, only a practical recommendation.

For the three hydrogen atoms in the ammonium group, we tested a systematic variation of the SIMU restraint in addition (see Section 2.2). However, with three different parameters to be modified for SIMU and no obvious correlations found, we refrained from presenting it in detail here. It is safe to assume that the default values are not problematic, as in the cases of ISOR, DELU and RIGU. We applied them and observed that they have a positive impact on the visual impression of the hydrogen atom ADPs.

We have also found that with data quality that is not of charge-density quality but more representative for average polypeptide structures, the approximately chemically equivalent N—H bonds in DDD are different by more than one σ after initial HAR and also differ from the reference neutron values by about the same amount. Therefore, here a SADI distance restraint is meaningful and was applied in the final DDD HAR models.

The water molecules in the 100 K structure were treated in the same way as the O—H bonds in the main peptide, and the hydrogen atoms could be refined anisotropically upon use of the discussed restraints. However, due to their disorder, the water molecules were more tricky to handle in the 8 K structure. The same combination of ADP restraints as for the main peptide chain did not avoid NPD ADPs. Therefore, the hydrogen atoms were treated only isotropically. Moreover, O—H bond distances differed much more from the reference neutron values than the O—H bonds in the carboxyl groups after initial HAR attempts, so that here the distance and angle restraints DFIX and DANG were used.

We remark again that all restraint σ’s discussed and recommended in this section were applied in the same way to the 100 K dataset as they were applied to the 8 K dataset from which they were originally derived. From this independent test, we expect that the values are generally useful for peptide, oligopeptide and possible protein datasets that are of similar quality.

## 4. Conclusions

The tripeptide l-Asp-l-Asp-l-Asp (DDD) bears four carboxyl groups and can as such form different zwitterionic forms that were predicted theoretically to show different turn structures and folding behaviour (Duitch *et al.*, 2012 ▸). Here, we show experimentally that in the crystal structure the zwitterionic form with the deprotonated carboxyl­ate group at the N-terminus exists. With three co-crystallized water molecules serving as strongly hydrogen-bonded bridges between the N- and C-terminus, DDD maximally bends into a folded structure. Across two different polymorphs, we found five different water networks, all yielding the same folded structure, with conformational differences only in the carb­oxy­lic acid/ carboxyl­ate groups that point towards the backend of the peptide chain. This highlights the importance of water as an active biomolecule in peptide (protein) structures and the necessity of describing water and hydrogen atoms as accurately and precisely as possible in X-ray diffraction refinements.

For this purpose, here we use Hirshfeld atom refinement (HAR), which is known to describe hydrogen atoms correctly based on X-ray data (Woińska *et al.*, 2016 ▸). However, until now hydrogen-atom restraints have not been accessible to HAR, so we activated them in *olex2.refine* for use with *NoSpherA2*. We tested whether the default values of the ADP restraints ISOR, DELU, RIGU and SIMU found for non-hydrogen atoms in IAM also apply for hydrogen atoms in HAR. We found that there is no significant correlation between the values of the restraints and the *X*—H bond distances. Physically reasonable ADPs are not necessarily accurate ADPs, however, if physically reasonable ADPs are available, they should be preferred over NPD or isotropic ones. Hence, we suggest the following values for hydrogen-atom ADP restraints, although we note that many more chemical systems would need to be studies for a broad validity of the recommended values.

For ISOR, we recommend a value between 0.01 and 0.02 Å^2^, which is ten times more restrictive than the default value for non-hydrogen atoms. For DELU, we suggest the default value of 0.01 Å^2^ for both 1,2- and 1,3-restraints. For SIMU, we also suggest the default value of 0.08 Å^2^, however, this was not tested as rigorously as for the other restraints. For RIGU, the default values seem to be unnecessarily strict for hydrogen atoms, so that we suggest 0.008 to 0.01 Å^2^ for both 1,2- and 1,3-restraints. These conclusions hold for the refinement of X-ray data with hydrogen atoms in bonding environments that occur in peptides; other bonding types or radiation sources (electron diffraction) have not been tested.

We noted in the introduction that DELU and RIGU restraints are, strictly speaking, not suitable for hydrogen atoms because the Hirshfeld test, on which the definition of these restraints is based, is valid only for atoms with similar atomic masses. The hydrogen atom has a much more pronounced vibrational component along the bond direction than the heavier atom, so that restraints similar to DELU/RIGU designed for hydrogen atoms should include different scales for the atom pair along the bond direction (see Lübben *et al.*, 2015 ▸, for a suggestion how different scales of the internal modes of different atoms can be taken into account by computation). In theory, this should improve the hydrogen-atom ADP shapes. In practice, this study agrees with previous findings (Woińska *et al.*, 2024 ▸; Malaspina *et al.*, 2020 ▸; Dittrich *et al.*, 2017 ▸) that there is little information in the data for refining hydrogen ADPs so that their shape does not matter for refining accurate hydrogen positions. This means that already isotropic hydrogen-atom refinement would be sufficient. Therefore, we conclude that developing new hydrogen-specific restraints is unnecessary. However, this means that when DELU- and RIGU-restrained hydrogen-atom ADPs are applied and look physically reasonable, they are still not physically meaningful. Moreover, adding quantum-chemically aided (scaled) ADPs derived from either cluster or fully periodic frequency calculations is so fast that these could be applied for normal quantum-crystallographic refinements in the future (see Munshi *et al.*, 2008 ▸, and more recent developments in dynamic quantum crystallography, Hoser & Madsen, 2017 ▸; Woińska *et al.*, 2024 ▸). They have a clearly defined physical basis, whereas the choice of restraint type and restraint σ values is somewhat arbitrary and empirical.

## 5. Related literature

The following references are cited in the supporting information: Thomas *et al.* (2018 ▸); Turner *et al.* (2014 ▸); Mackenzie *et al.* (2017 ▸).

## Supplementary Material

Crystal structure: contains datablock(s) global, DDD_trihydrate_8K, DDD_trihydrate_100K. DOI: 10.1107/S2052520625006110/pl5045sup1.cif

includes more structural details (asymmetric unit representations, hydrogen-bonding table), values of restraints used in the final models for both 8K and 100K structures, an overlay of archetype structures, a discussion of cohesive energies, and bond-length versus restraint-value plots for 1,3-restraint values. DOI: 10.1107/S2052520625006110/pl5045sup2.pdf

CCDC references: 2100425, 2388831

## Figures and Tables

**Table 1:** Crystallographic, measurement, and HAR refinement details For DDD trihydrate: C_12_H_17_N_3_O_10_·3H_2_O, *M*_r_ = 417.33, triclinic, *P*1, *Z* = 1, *F*(000) = 220.1.

	At 8 K	At 100 K
CCDC number	2100425	2388831
*a*, *b*, *c* (Å)	4.726 (3), 9.369 (4), 10.239 (4)	4.7910 (6), 9.4366 (12), 10.2881 (14)
α, β, γ (°)	88.796 (17), 85.14 (5), 78.90 (7)	88.648 (5), 84.639 (5), 80.234 (6)
*V* (Å^3^)	443.3 (4)	456.37 (10)
Density (calculated) (g cm^−3^)	1.563	1.518
μ (mm^−1^)	0.073	0.082
Crystal size (mm)	0.35 × 0.20 × 0.05	0.35 × 0.30 × 0.20
Radiation type, λ (Å)	Synchrotron, 0.51660	Synchrotron, 0.56000
No. of reflections collected	102398	16706
within the θ (°) limit	1.45 to 26.95	3.13 to 38.54
Resolution max (Å)	0.57	0.45
Completeness (%)	93	73.6†
No. of independent reflections	9270	15225
Redundancy	11.1	1.1
*R*_int_	0.062	0.013
No. of parameters, restraints	456, 178	480, 261
No. of observed reflections [*I* > 2σ(*I*)]	9204	14647
Final *R* indices [*I* > 2σ(*I*)] (HAR)		
*R*1	0.0387	0.0269
*wR*2	0.1009	0.0695
Goodness-of-fit	1.113	1.111
Δρ_max_, Δρ_min_ (e Å^−3^)	0.741, −0.566	0.372, −0.352

Computer programs: *NoSpherA2* (Kleemiss *et al.*, 2021 ▸), *olex2.refine* (Bourhis *et al.*, 2015 ▸), *OLEX2* (Dolomanov *et al.*, 2009 ▸).

† A second crystallite was identified in the diffraction images. Since *XDS* does not allow integration with a twin mask, the structure factor file was detwinned manually after the integration by removing all overlapped reflections. This led to the low completeness.
