# Supplementary material for: Role of restraints on hydrogen atoms in Hirshfeld atom refinement: the case of tri-aspartic acid trihydrate
Source: Acta Crystallogr B Struct Sci Cryst Eng Mater. 2025 Sep 5;81(Pt 5):484–97. doi: 10.1107/S2052520625006110 (PMC12517054; doi:10.1107/S2052520625006110)
Supplement: Supplementary file 2 [file b-81-00484-sup2.pdf]

Supporting Information for

Role of restraints on hydrogen atoms in Hirshfeld Atom Refinement: the case of tri-aspartic acid trihydrate

Ravish Sankolli[a], Lorraine A. Malaspina[a*], Oleg V. Dolomanov[b], Peter Luger[c], Julian J. Holstein[d], Carsten Paulmann[e], Wolfgang Morgenroth[f], Florian Kleemiss[g], Birger Dittrich[hi], and Simon Grabowsky[a*]

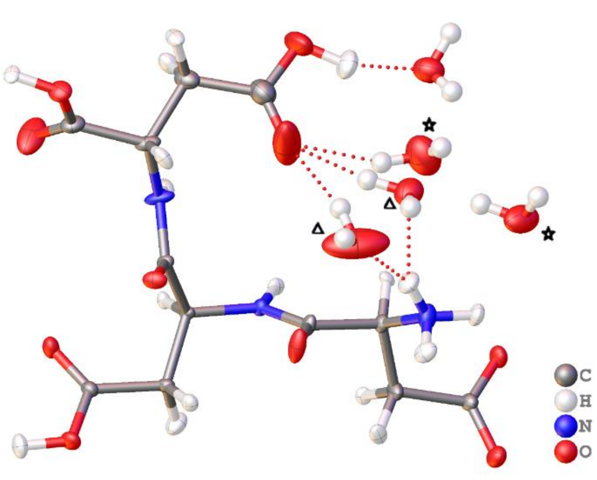

(a)

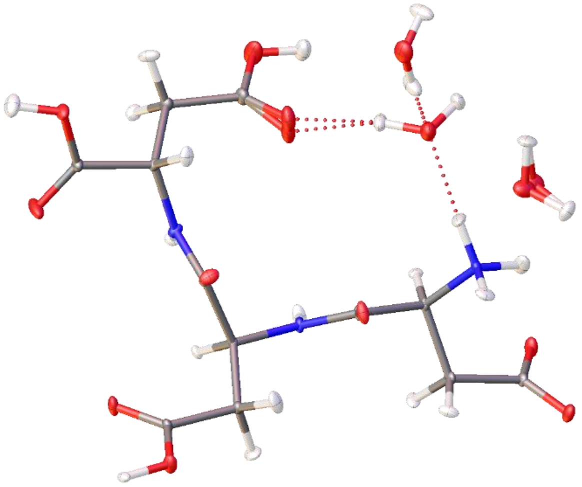

(b)

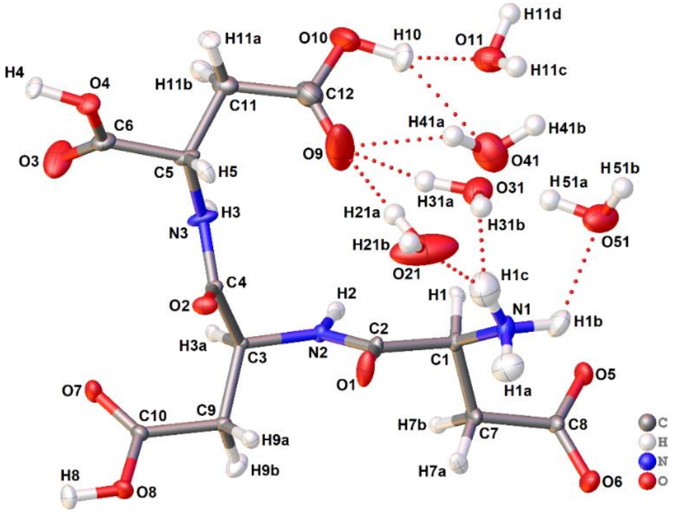

(c)

Figure S1. Asymmetric units of the HAR-refined structures of tri-aspartic acid trihydrate at 8K (a) and (c) 100K (b). In (a), the two pairs of mutually exclusive disordered water molecules are denoted with signs (pair ∗ with refined occupancies 0.13/0.87 (O41/O51), and pair Δ with 0.25/0.75 (O21/O31)). In (c), all atom labels are given. All ADPs at a 50% probability level, isotropic ones at arbitrary scale. Pictures generated with Olex2.

Table S1. Restraints used in the final models

8K structure	100K structure
ISOR 0.1 0.02 H3<br>ISOR 0.1 0.01 H8<br>ISOR 0.1 0.01 H3A<br>ISOR 0.1 0.01 H7B<br>ISOR 0.1 0.01 H9B<br>ISOR 0.1 0.02 H11B H11A<br>ISOR 0.1 0.01 H9A<br>ISOR 0.1 0.01 H2<br>ISOR 0.1 0.01 H1<br>ISOR 0.1 0.02 H5<br>ISOR 0.1 0.02 H7A<br>ISOR 0.1 0.02 H4<br>ISOR 0.1 0.02 H10<br>ISOR 0.1 0.02 H1A<br>ISOR 0.1 0.02 H1B<br>ISOR 0.1 0.02 H1C<br>DELU 0.01 0.02 C3 H3A<br>RIGU 0.008 0.008 C7 H7B<br>RIGU 0.008 0.008 N2 H2<br>RIGU 0.008 0.008 C3 H3A<br>RIGU 0.008 0.008 C9 H9A H9B<br>RIGU 0.008 0.008 O4 H4 O8 H8 O10 H10<br>RIGU 0.008 0.008 N1 H1A N1 H1B N1 H1C<br>RIGU 0.008 0.008 C11 H11A H11B<br>SADI N1 H1A N1 H1B N1 H1C<br>DANG 1.518 0.001 H31A H31B<br>DANG 1.518 0.001 H21A H21B<br>DANG 1.518 0.001 H41A H41B<br>DANG 1.518 0.001 H51A H51B<br>DANG 1.518 0.001 H11C H11D<br>DFIX 0.96 0.001 O51 H51A O51 H51B<br>DFIX 0.96 0.001 O41 H41A O41 H41B<br>DFIX 0.96 0.001 O21 H21A O21 H21B<br>DFIX 0.96 0.001 O31 H31A O31 H31B<br>DFIX 0.96 0.001 O11 H11C O11 H11D	ISOR 0.1 0.01 H4 H5 H11d H1c H3 H9a H1<br>ISOR 0.1 0.01 H31a O31a O31 H31b<br>ISOR 0.1 0.01 H1c<br>ISOR 0.1 0.01 H8<br>ISOR 0.1 0.02 H3<br>ISOR 0.1 0.02 O9a O9<br>ISOR 0.1 0.01 H7a<br>ISOR 0.1 0.01 H5<br>ISOR 0.1 0.02 H9b<br>ISOR 0.1 0.01 H11a H11b<br>ISOR 0.1 0.01 H10 H21a<br>ISOR 0.1 0.01 H2<br>DELU 0.001 0.001 O9a O9 C12<br>DELU 0.01 0.01 H31a O31a H31b<br>DELU 0.01 0.01 H31a O31 H31b<br>RIGU 0.004 0.004 H7a C7<br>RIGU 0.004 0.004 H7b C7<br>RIGU 0.004 0.004 H11a C11 H11b<br>RIGU 0.004 0.004 N1 H1a H1b H1c<br>RIGU 0.004 0.004 H1 C1<br>RIGU 0.004 0.004 H8 O8<br>RIGU 0.008 0.008 H7b<br>RIGU 0.004 0.004 H10 O10<br>RIGU 0.008 0.008 H11d O11<br>RIGU 0.004 0.004 H3 N3<br>RIGU 0.004 0.004 H4 O4<br>RIGU 0.004 0.004 H31b O31 H31a<br>RIGU 0.004 0.004 H31b O31a H31a<br>RIGU 0.004 0.004 H2 N2<br>RIGU 0.004 0.004 H21b O21 H21a<br>SADI 0.002 C1 H1 C5 H5<br>SADI 0.002 O4 H4 O10 H10 O8 H8<br>SADI 0.002 O21 H21a O21 H21b<br>SADI 0.002 O31a H31b O31 H31b O31 H31a O31a H31a<br>SIMU 0.0002 0.0002 2 O31 O31a<br>DANG 1.524 0.001 H31a H31b<br>DFIX 1.092 0.002 C9 H9a C9 H9b C11 H11b C11 H11a C7 H7b C7 H7a

Table S2. Selection of strongest hydrogen bonds. Distance d in Å, angle < in °. D = donor atom, H = hydrogen atom, A = acceptor atom. All hydrogen atom positions were refined during HAR of both data sets.

D–H···A	d(D–H)	d(H···A)	d(D···A)	<(D–HA)	Symmetry
**8K:**					
N1–H1A···O5	0.97(2)	1.91(3)	2.805(2)	152(2)	x+1,y,z
N1–H1B···O7	1.01(2)	1.91(2)	2.856(2)	154(3)	x,y,z-1
N1–H1C···O31	0.94(3)	1.84(3)	2.703(2)	151(3)	--
N2–H2···O1	0.989(19)	1.978(19)	2.922(2)	158.8(16)	-1+x,y,z
N3–H3···O2	0.97(3)	1.97(3)	2.901(2)	159(2)	-1+x,y,z
O4–H4···O6	1.00(2)	1.53(2)	2.5284(19)	173(3)	x,1+y,1+z
O8–H8···O5	0.97(2)	1.67(2)	2.632(2)	174(2)	1+x,y,1+z
O10–H10···O11	1.07(3)	1.55(3)	2.600(2)	166(3)	--
O11–H11D···O6	0.96(3)	1.76(2)	2.699(2)	167(2)	-1+x,1+y,z
O31–H31A···O9	0.960(13)	1.701(17)	2.645(2)	167(2)	--
O51–H51B···O3	0.96(2)	1.94(2)	2.857(2)	159.3(15)	x,y,-1+z
**100K:**					
N1–H1A···O5	1.007(14)	1.959(14)	2.8177(6)	141.5(12)	x+1,y,z
N1–H1B···O7	1.070(14)	1.808(13)	2.8511(6)	163.9(12)	x,y,z-1
N1–H1C···O21	1.052(18)	1.798(17)	2.8033(6)	158.0(2)	--
N2–H2···O1	1.007(15)	2.041(14)	2.9813(6)	154.5(12)	-1+x,y,z
N3–H3···O2	0.950(15)	2.011(15)	2.9231(6)	160.2(13)	-1+x,y,z
O4–H4···O6	0.979(14)	1.575(12)	2.5310(6)	164.5(17)	x,1+y,1+z
O8–H8···O5	0.976(6)	1.696(7)	2.6675(6)	172.9(13)	1+x,y,1+z
O10–H10···O11	0.975(5)	1.614(8)	2.5766(7)	168.8(18)	-1+x,y,z
O11–H11C···O6	0.898(16)	1.961(18)	2.7690(7)	149.0(17)	x,1+y,z
O11–H11D···O21	0.903(18)	1.900(19)	2.7846(7)	165.9(18)	--
O21–H21A···O9	0.961(12)	1.815(10)	2.7398(17)	160.5(13)	--
O21–H21B···O3	0.956(12)	1.942(11)	2.8639(7)	161.3(12)	x,y,-1+z
O31–H31A···O3	0.997(7)	1.918(8)	2.9020(8)	168.9(8)	--
O31–H31B···O7	0.993(8)	2.040(9)	2.9127(7)	145.6(7)	--

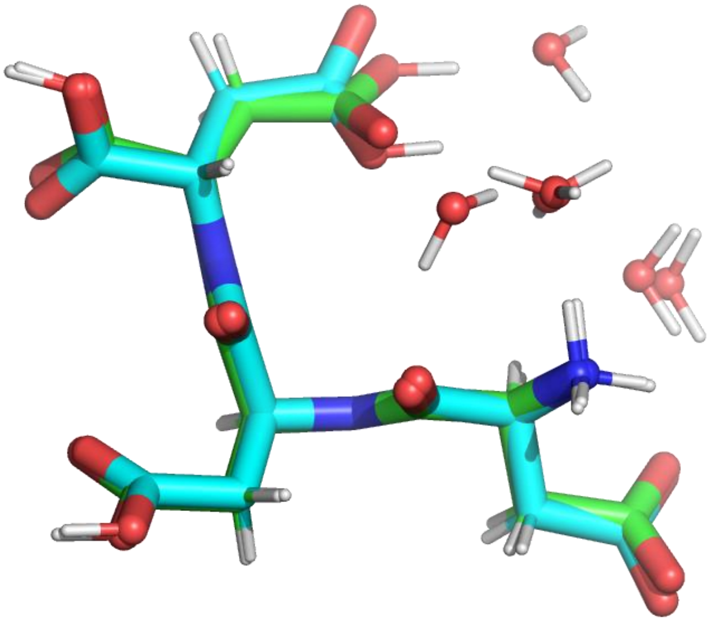

Figure S2. Overlay of the two molecule-in-crystal optimized archetype structures of DDD at 8K. They represent two different local energy minima. The differences in conformation of the tripeptide and the water positions coincide with the experimentally found disordered crystal structure.

To further investigate the energetic differences caused by the different hydrogen-bonding patterns, we estimated the cohesive energy of the DDD zwitterionic molecule in each of the four slightly different environments depicted in Figure 3 (main manuscript) according to a procedure described in Thomas *et al.*, 2018 (references given in the main manuscript). This method makes use of scaled total model interaction energies (Turner *et al.*, 2014; Mackenzie *et al.*, 2017) calculated within the software CrystalExplorer (Spackman *et al.*, 2021). Since the three water molecules of the asymmetric unit were included in the calculations only as interaction partners of the DDD molecule, the four different estimated DDD cohesive energies are not identical to the lattice energy of the crystal, but allow a quantification of the energy difference produced by the different water positions. The cohesive energies are -438.6 kJ/mol (with O51 and O21 present), -430.3 kJ/mol (with O41 and O31 present), -454.9 kJ/mol (with O41 and O21 present), and -422.0 kJ/mol (with O51 and O31 present). This means that the maximum energy difference is about 30 kJ/mol, which is significant, but obviously not significant enough to change the conformation of the DDD zwitterion as it is not disordered in the crystal structure. The lowest energy corresponds to O41 and O21 being present simultaneously, which seems to agree with the strongest bridging interactions as depicted in Figure 3. However, surprisingly the main disorder component (O51 and O31 present simultaneously, see caption of Figure 2) gives rise to the smallest cohesive energy. On the other hand, as expected for a mainly hydrogen-bond stabilized crystal, in all cases electrostatic energy components clearly dominate over polarization and dispersion (see below).

Table S3. Scale factors for the calculation of total energies from model energies.

Energy Model	k_ele	k_pol	k_disp	k_rep
CE-B3LYP ... B3LYP/6-31G(d,p) electron densities	1.057	0.740	0.871	0.618

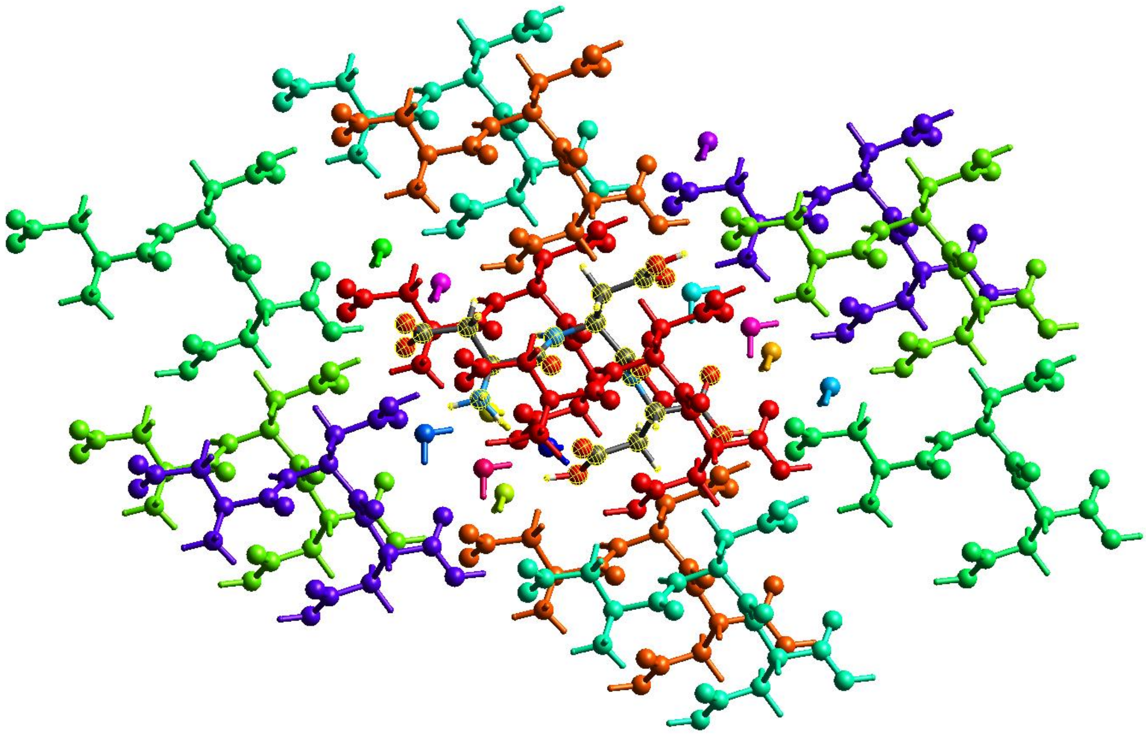

Figure S3. Cluster of molecules used for the calculation of the cohesive energy for the situation with water molecules O51 and O21 present. The symmetry-indepenent DDD molecule is highlighted with a yellow net. All other molecules are color-coded according to their symmetry operation, see Table S4.

Table S4. Dimer interaction energies between the central symmetry-independent DDD molecule and its interaction partners (R = distance between centers of mass of both partners in Å) for the situation with water molecules O51 and O21 present. Color code shown in Figure S3. All energies in kJ/mol. The energy components are given unscaled, whereas the total energy is the sum of scaled components (Table S3 for scale factors). The cohesive energy of the DDD molecule is the sum of all total energies, including the repetition factor N, divided by 2 (-438.6 kJ/mol). Calculated at the B3LYP/6-31G(d,p) level of theory.

	N	R	E_ele	E_pol	E_dis	E_rep	E_tot
	2	4.73	-119.1	-62.0	-73.3	137.1	-151.0
	2	9.65	-8.4	-3.2	-21.5	14.0	-21.4
	1	7.46	-3.7	-3.4	-4.5	15.5	-0.8
	1	4.16	4.9	-4.3	-11.9	12.9	-0.5
	1	5.45	-95.4	-21.7	-6.8	109.4	-55.3
	2	11.63	-84.0	-27.1	-7.7	74.1	-69.8

	1	7.74	-27.0	-4.6	-6.3	5.6	-33.9
	2	14.02	-111.9	-33.9	-7.6	112.0	-80.8
	2	9.37	-16.7	-4.2	-19.5	10.2	-31.5
	1	5.12	-20.4	-5.9	-9.0	31.1	-14.6
	1	8.27	8.3	-0.7	-1.6	0.2	7.0
	1	5.73	18.7	-8.2	-9.9	11.7	12.3
	1	3.42	-77.0	-23.8	-14.0	82.2	-60.5
	2	10.24	-46.9	-23.5	-16.5	37.0	-58.5
	1	8.79	3.4	-0.4	-1.0	0.0	2.4
	1	6.88	-62.8	-20.3	-8.0	63.9	-48.9
	1	5.99	2.2	-1.2	-3.4	0.4	-1.2
	1	5.85	-11.1	-6.7	-7.5	6.2	-19.3

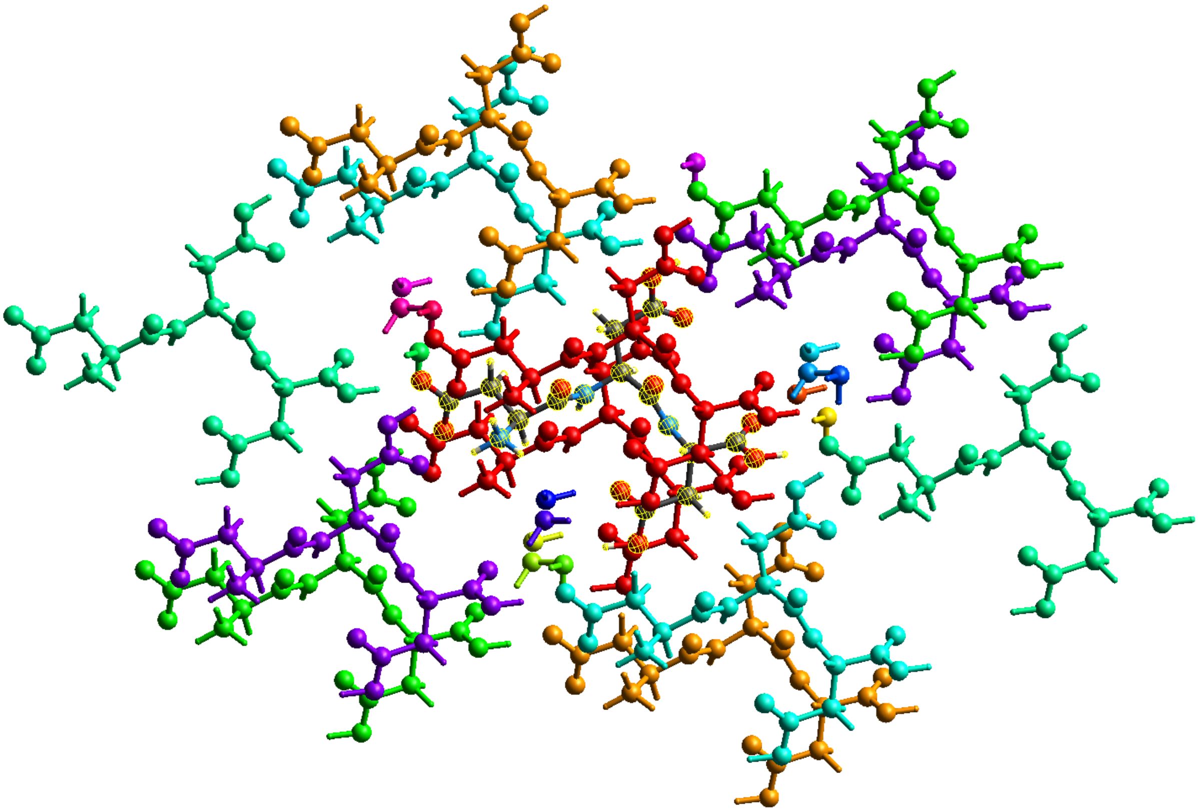

Figure S4. Cluster of molecules used for the calculation of the cohesive energy for the situation with water molecules O41 and O31 present. The symmetry-indepenent DDD molecule is highlighted with a yellow net. All other molecules are color-coded according to their symmetry operation, see Table S5.

Table S5. Dimer interaction energies between the central symmetry-independent DDD molecule and its interaction partners (R = distance between centers of mass of both partners in Å) for the situation with water molecules O41 and O31 present. Color code shown in Figure S4. All energies in kJ/mol. The energy components are given unscaled, whereas the total energy is the sum of scaled components (Table S3 for scale factors). The cohesive energy of the DDD molecule is the sum of all total energies, including the repetition factor N, divided by 2 (-430.3 kJ/mol). Calculated at the B3LYP/6-31G(d,p) level of theory.

	N	R	E_ele	E_pol	E_dis	E_rep	E_tot
	2	4.73	-119.1	-62.0	-73.3	137.1	-151.0
	1	7.66	-4.9	-0.3	-0.7	0.0	-6.0
	2	9.65	-8.4	-3.2	-21.5	14.0	-21.4
	1	7.46	-3.7	-3.4	-4.5	15.5	-0.8
	1	5.77	16.6	-2.0	-2.6	0.2	13.9
	1	7.03	6.5	-0.5	-0.6	0.0	6.0
	1	5.45	-95.4	-21.7	-6.8	109.4	-55.3
	2	11.63	-84.0	-27.1	-7.7	74.1	-69.8
	1	7.74	-27.0	-4.6	-6.3	5.6	-33.9
	2	14.02	-111.9	-33.9	-7.6	112.0	-80.8
	2	9.37	-16.7	-4.2	-19.5	10.2	-31.5
	1	7.04	11.2	-1.2	-2.5	1.7	9.8
	1	6.86	-31.0	-7.2	-4.4	57.8	-6.2
	1	8.27	8.3	-0.7	-1.6	0.2	7.0
	1	4.00	-102.9	-29.7	-13.7	120.4	-68.4
	1	4.32	-48.5	-8.6	-13.2	41.6	-43.5
	2	10.24	-46.9	-23.5	-16.5	37.0	-58.5
	1	8.79	3.4	-0.4	-1.0	0.0	2.4
	1	8.33	9.4	-1.7	-1.8	0.4	7.4
	1	8.44	-15.5	-2.6	-2.5	0.6	-20.1
	1	6.88	-62.8	-20.3	-8.0	63.9	-48.9

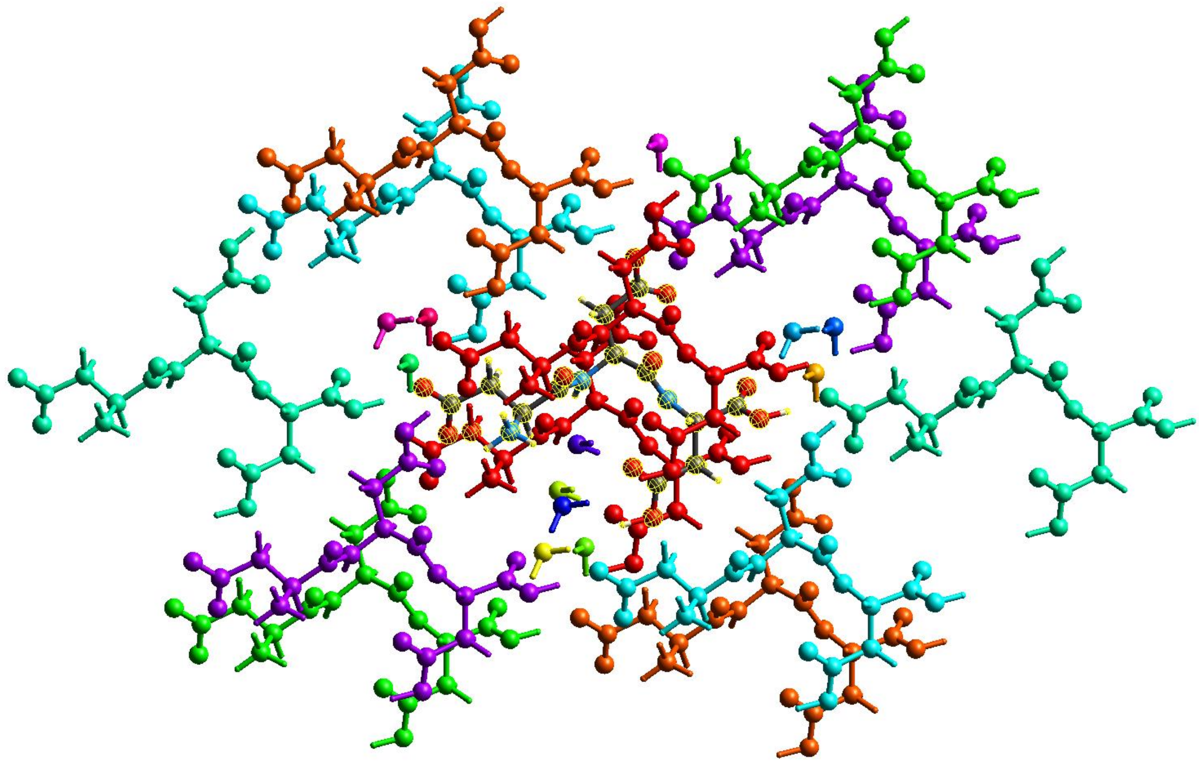

Figure S5. Cluster of molecules used for the calculation of the cohesive energy for the situation with water molecules O41 and O21 present. The symmetry-indepenent DDD molecule is highlighted with a yellow net. All other molecules are color-coded according to their symmetry operation, see Table S6.

Table S6. Dimer interaction energies between the central symmetry-independent DDD molecule and its interaction partners (R = distance between centers of mass of both partners in Å) for the situation with water molecules O41 and O21 present. Color code shown in Figure S5. All energies in kJ/mol. The energy components are given unscaled, whereas the total energy is the sum of scaled components (Table S3 for scale factors). The cohesive energy of the DDD molecule is the sum of all total energies, including the repetition factor N, divided by 2 (-454.9 kJ/mol). Calculated at the B3LYP/6-31G(d,p) level of theory.

	N	R	E_ele	E_pol	E_dis	E_rep	E_tot
	2	4.73	-119.1	-62.0	-73.3	137.1	-151.0
	2	9.65	-8.4	-3.2	-21.5	14.0	-21.4
	1	7.46	-3.7	-3.4	-4.5	15.5	-0.8
	1	7.03	6.5	-0.5	-0.6	0.0	6.0
	1	4.16	4.9	-4.3	-11.9	12.9	-0.5
	1	5.45	-95.4	-21.7	-6.8	109.4	-55.3
	2	11.63	-84.0	-27.1	-7.7	74.1	-69.8
	1	7.74	-27.0	-4.6	-6.3	5.6	-33.9
	2	14.02	-111.9	-33.9	-7.6	112.0	-80.8

	2	9.37	-16.7	-4.2	-19.5	10.2	-31.5
	1	6.86	-31.0	-7.2	-4.4	57.8	-6.2
	1	8.27	8.3	-0.7	-1.6	0.2	7.0
	1	4.32	-48.5	-8.6	-13.2	41.6	-43.5
	1	3.42	-77.0	-23.8	-14.0	82.2	-60.5
	2	10.24	-46.9	-23.5	-16.5	37.0	-58.5
	1	8.79	3.4	-0.4	-1.0	0.0	2.4
	1	8.44	-15.5	-2.6	-2.5	0.6	-20.1
	1	6.88	-62.8	-20.3	-8.0	63.9	-48.9

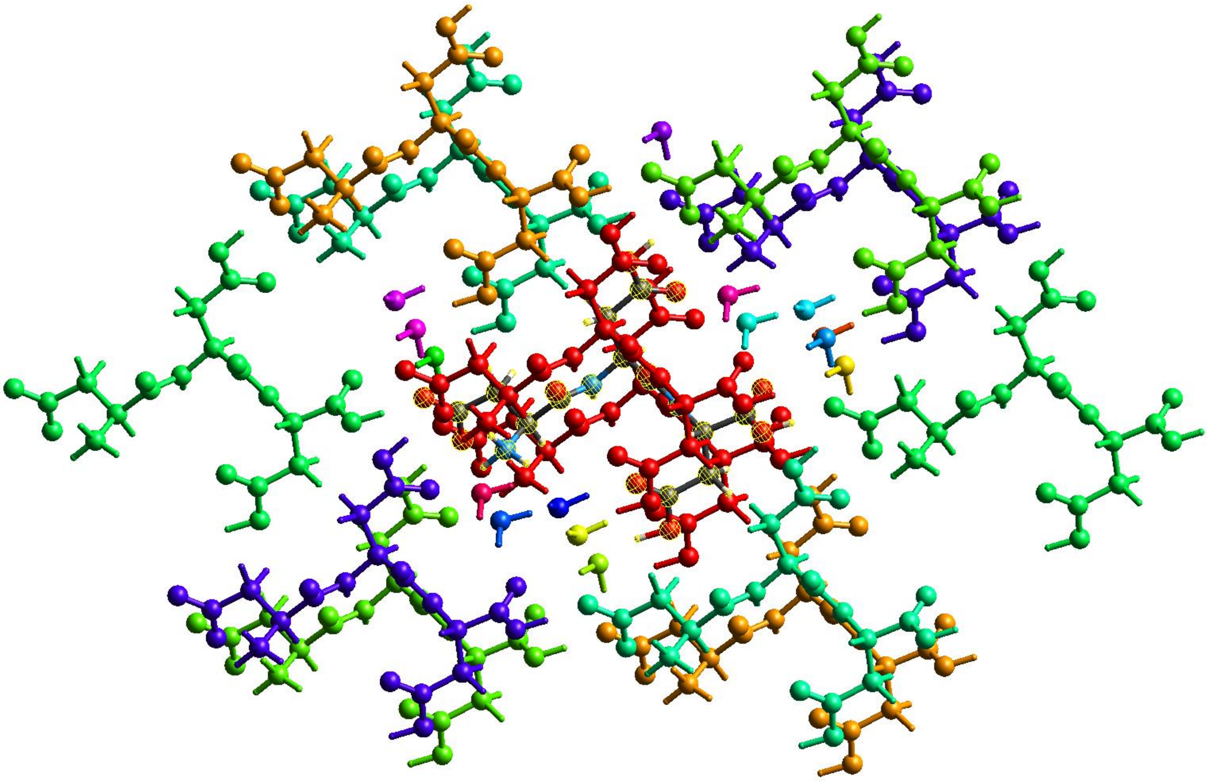

Figure S6. Cluster of molecules used for the calculation of the cohesive energy for the situation with water molecules O51 and O31 present. The symmetry-indepenent DDD molecule is highlighted with a yellow net. All other molecules are color-coded according to their symmetry operation, see Table S7.

Table S7. Dimer interaction energies between the central symmetry-independent DDD molecule and its interaction partners (R = distance between centers of mass of both partners in Å) for the situation with water molecules O51 and O31 present. Color code shown in Figure S6. All energies in kJ/mol. The energy components are given unscaled, whereas the total energy is the sum of scaled components (Table S3 for scale factors). The cohesive energy of the DDD molecule is the sum of all total energies, including the repetition factor N, divided by 2 (-422.0 kJ/mol). Calculated at the B3LYP/6-31G(d,p) level of theory.

	N	R	E_ele	E_pol	E_dis	E_rep	E_tot
	2	4.73	-119.1	-62.0	-73.3	137.1	-151.0
	1	7.66	-4.9	-0.3	-0.7	0.0	-6.0
	2	9.65	-8.4	-3.2	-21.5	14.0	-21.4
	1	7.46	-3.7	-3.4	-4.5	15.5	-0.8
	1	5.77	16.6	-2.0	-2.6	0.2	13.9
	1	5.45	-95.4	-21.7	-6.8	109.4	-55.3
	2	11.63	-84.0	-27.1	-7.7	74.1	-69.8
	1	7.74	-27.0	-4.6	-6.3	5.6	-33.9
	2	14.02	-111.9	-33.9	-7.6	112.0	-80.8
	2	9.37	-16.7	-4.2	-19.5	10.2	-31.5
	1	5.12	-20.4	-5.9	-9.0	31.1	-14.6
	1	7.04	11.2	-1.2	-2.5	1.7	9.8
	1	8.27	8.3	-0.7	-1.6	0.2	7.0
	1	5.73	18.7	-8.2	-9.9	11.7	12.3
	1	4.00	-102.9	-29.7	-13.7	120.4	-68.4
	2	10.24	-46.9	-23.5	-16.5	37.0	-58.5
	1	8.79	3.4	-0.4	-1.0	0.0	2.4
	1	8.33	9.4	-1.7	-1.8	0.4	7.4
	1	6.88	-62.8	-20.3	-8.0	63.9	-48.9
	1	5.99	2.2	-1.2	-3.4	0.4	-1.2
	1	5.85	-11.1	-6.7	-7.5	6.2	-19.3

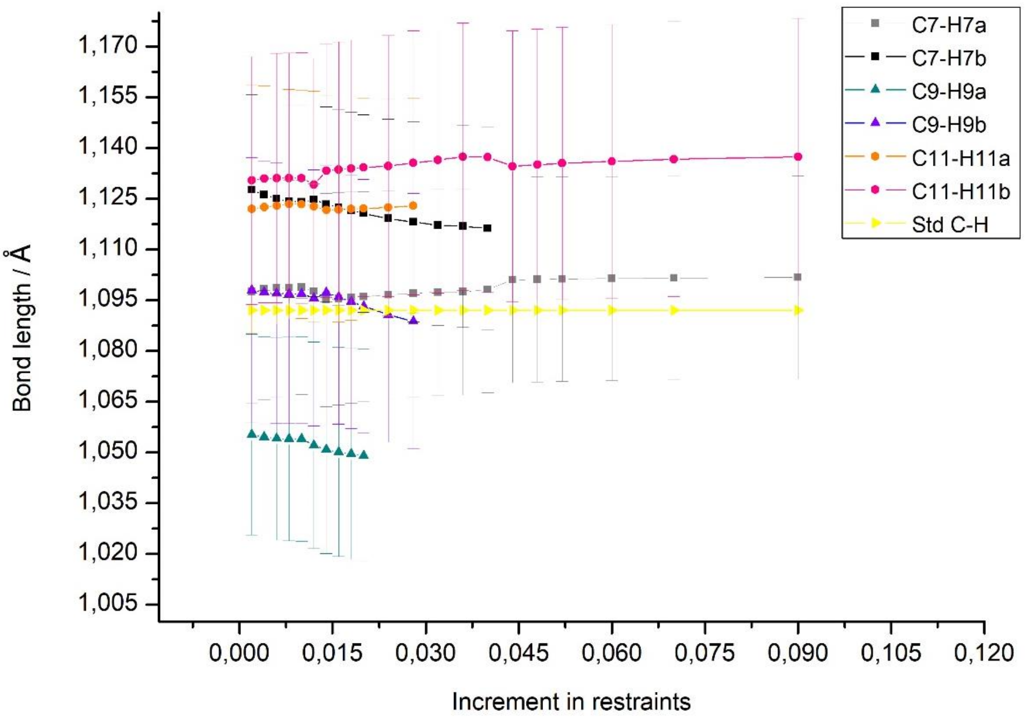

Figure S7. Refined C-H bond lengths (Å) vs. ISOR restraint σ's (Å^2) for methylene $–CH_2$ functional groups. Vertical bars are the σ's upon least-squares refinement. The respective reference C-H bond distance from neutron diffraction (Allen & Bruno, 2010) is given as yellow data points (Std C-H).

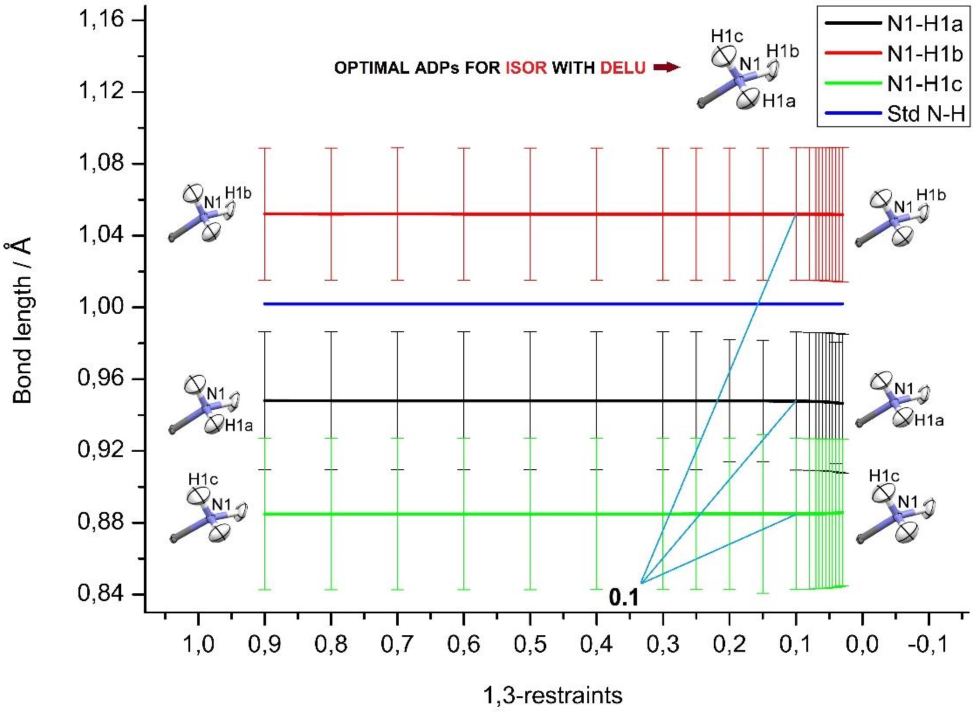

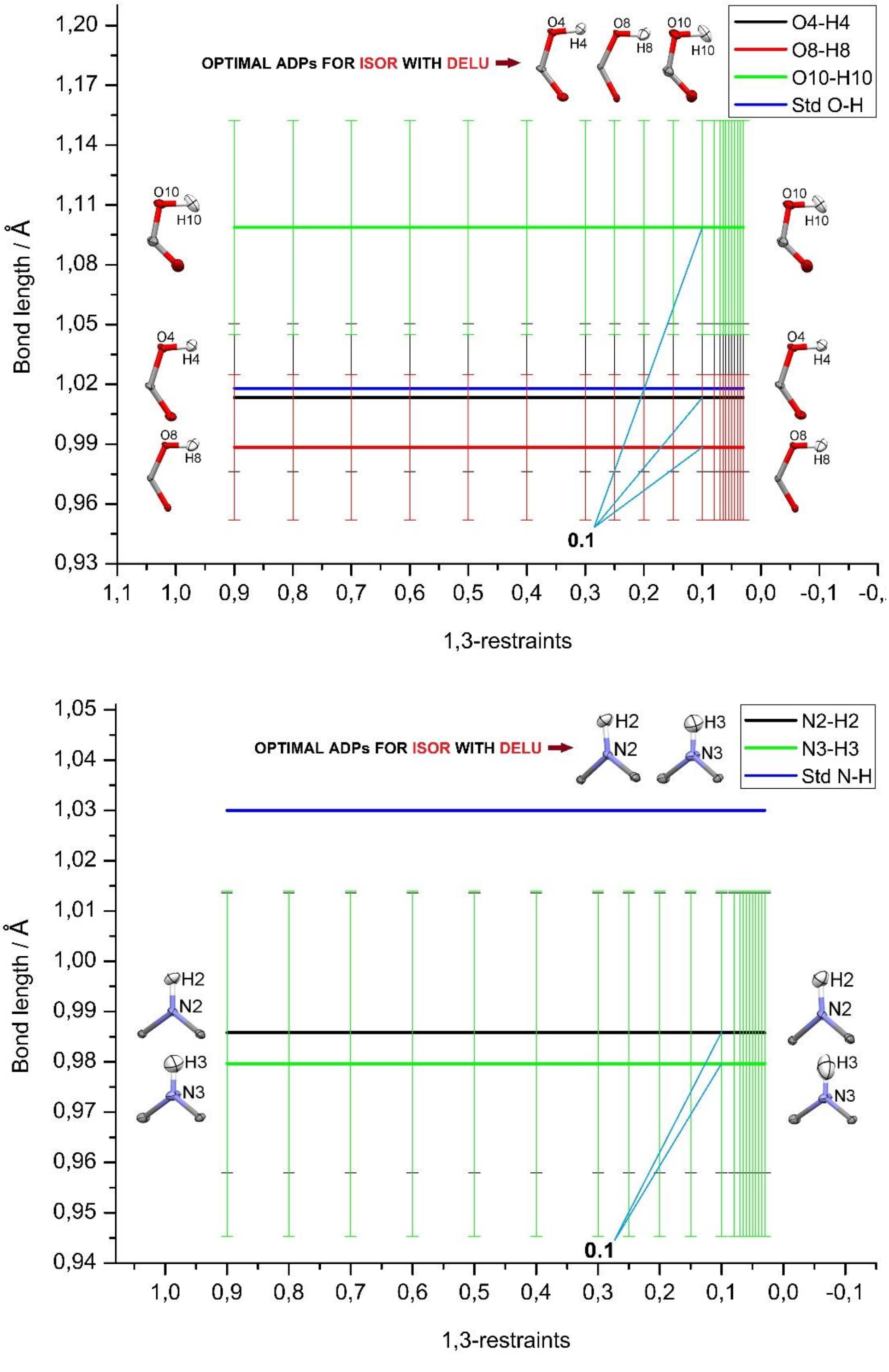

Figure S8. Refined X-H bond lengths (Å) vs. DELU 1,3-restraint σ's ($Å^2$) for –$NH3^+$, –COOH, and –NH functional groups, under a mild ISOR restraint. The respective reference X-H bond distances from neutron diffraction (Allen & Bruno, 2010) are given as blue lines. The visual appearance of the ADPs for the two extreme choices of DELU σ's are given as inserts in the plots. The values chosen as optimum compromise between flexibility and acceptable shape of the ADPs are given in the plots (in $Å^2$), and the corresponding ADP shapes above the plots. Similar plots for the DELU 1,2-restraint σ's are given in Figure 5. Comparison between Figure 5 and S8 shows that the choice of the 1,3-restraint value is irrelevant for the refined bond length, and has only a small effect on the ADP shape on its own.

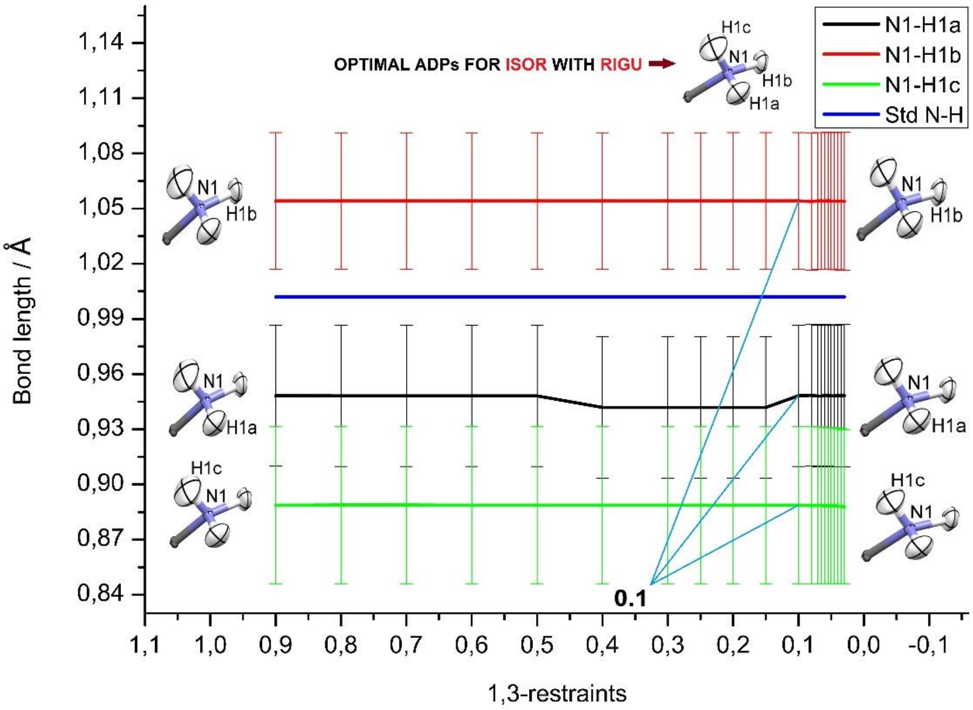
N1-H1a
N1-H1b
N1-H1c
Std N-H
OPTIMAL ADPs FOR ISOR WITH RIGU
H1c
N1
H1b
H1a
0.1
Bond length / Å
1,3-restraints

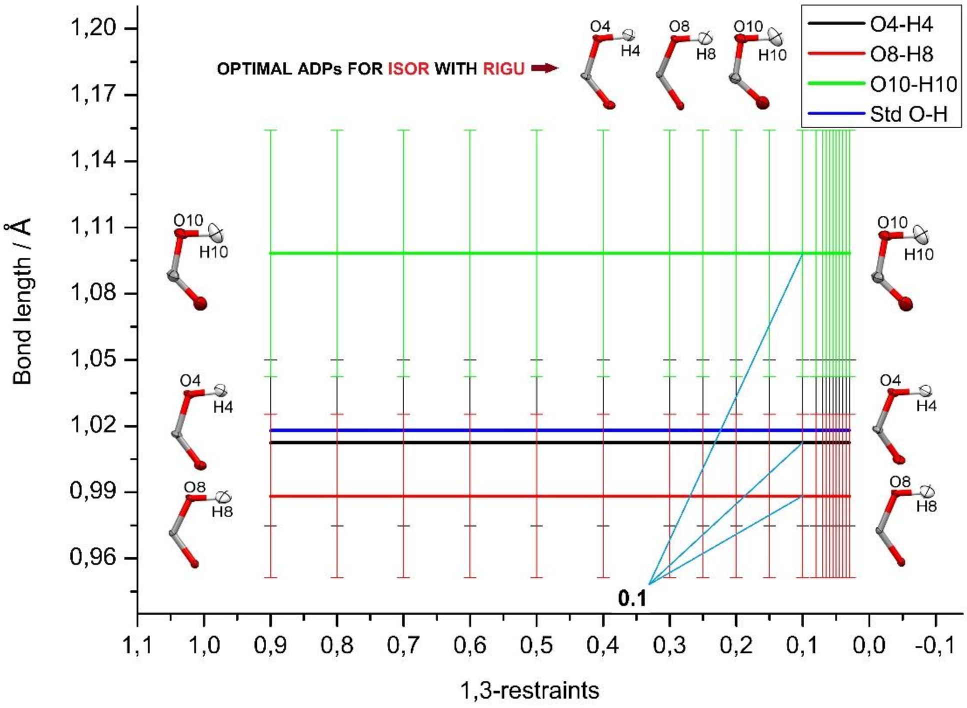
O4-H4
O8-H8
O10-H10
Std O-H
OPTIMAL ADPs FOR ISOR WITH RIGU
O4
H4
O8
H8
O10
H10
0.1
Bond length / Å
1,3-restraints

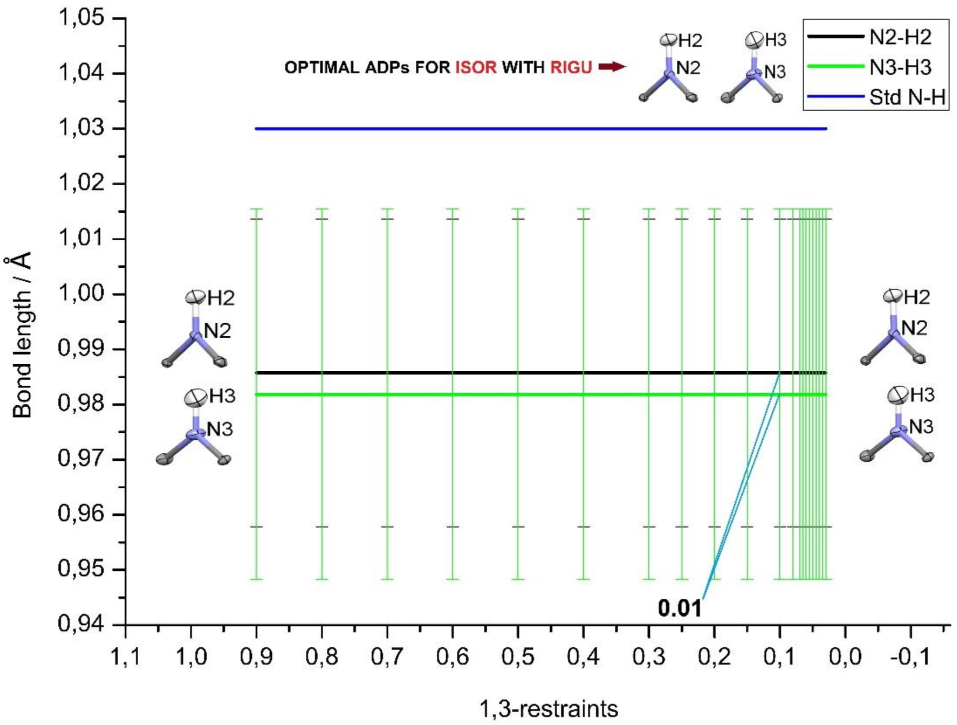

Figure S9. Refined X-H bond lengths (Å) vs. RIGU 1,3-restraint σ's (Å^2) for –NH3$^+$, –COOH, and –NH functional groups, under a mild ISOR restraint. The respective reference X-H bond distances from neutron diffraction (Allen & Bruno, 2010) are given as blue lines. The visual appearance of the ADPs for the two extreme choices of RIGU σ's are given as inserts in the plots. The values chosen as optimum compromise between flexibility and acceptable shape of the ADPs are given in the plots (in Å^2), and the corresponding ADP shapes above the plots. Similar plots for the RIGU 1,3-restraint σ's are given in Figure 6. Comparison between Figure 6 and S9 shows that the choice of the 1,3-restraint value is irrelevant for both the refined bond length and the ADP shape on its own.
